# A genomic island integrated into *recA* of *Vibrio cholerae* contains a divergent *recA* and provides multi-pathway protection from DNA damage

**DOI:** 10.1111/1462-2920.12512

**Published:** 2014-06-26

**Authors:** Rita A Rapa, Atiqul Islam, Leigh G Monahan, Ankur Mutreja, Nicholas Thomson, Ian G Charles, Harold W Stokes, Maurizio Labbate

**Affiliations:** 1 ithree Institute, University of TechnologyPO Box 123 Broadway, Sydney, NSW, 2007, Australia; 2 Department of Medical and Molecular Biosciences, University of TechnologySydney, NSW, Australia; 3 Wellcome Trust Sanger InstituteCambridge, UK

## Abstract

Lateral gene transfer (LGT) has been crucial in the evolution of the cholera pathogen, *Vibrio cholerae*. The two major virulence factors are present on two different mobile genetic elements, a bacteriophage containing the cholera toxin genes and a genomic island (GI) containing the intestinal adhesin genes. Non-toxigenic *V. cholerae* in the aquatic environment are a major source of novel DNA that allows the pathogen to morph via LGT. In this study, we report a novel GI from a non-toxigenic *V. cholerae* strain containing multiple genes involved in DNA repair including the recombination repair gene *recA* that is 23% divergent from the indigenous *recA* and genes involved in the translesion synthesis pathway. This is the first report of a GI containing the critical gene *recA* and the first report of a GI that targets insertion into a specific site within *recA*. We show that possession of the island in *Escherichia coli* is protective against DNA damage induced by UV-irradiation and DNA targeting antibiotics. This study highlights the importance of genetic elements such as GIs in the evolution of *V. cholerae* and emphasizes the importance of environmental strains as a source of novel DNA that can influence the pathogenicity of toxigenic strains.

## Introduction

*Vibrio cholerae* is a common inhabitant of marine and estuarine waters and is the causative agent of the diarrheal disease cholera. Although there are over 200 O-antigen serogroups among *V. cholerae* strains, only two, O1 and O139, are known to cause pandemics of cholera disease (Kaper *et al*., 1995). Lateral gene transfer (LGT) has largely contributed to the emergence of new pandemic strains of cholera (Faruque and Mekalanos, 2003; Keymer and Boehm, 2011). The appearance of the O139 serogroup and the so-called hybrid strains in the early 1990s are prime examples (Ramamurthy *et al*., 2003; Safa *et al*., 2009). Mobile genetic elements (MGEs) have been pivotal in the evolution of *V. cholerae* including diverse elements, such as the genomic islands (GIs) VPI-1, VPI-2, VSP-1, VSP-2, an integrative conjugative element, and the bacteriophage CTX (Faruque and Mekalanos, 2003; Grim *et al*., 2010). GIs are defined as large chromosomal regions that have features suggestive of recent LGT (Boyd *et al*., 2008). They have the capacity to excise and form circular intermediates and often target tRNA loci for their integration. In *V. cholerae*, GIs have been implicated in causing human disease and in environmental survival. For example, the replacement of the O1 classical biotype by the O1 El Tor biotype in the 1960s is suggested to be due to the acquisition of VSP-1 and VSP-2 that have probably enhanced epidemic spread (Faruque and Mekalanos, 2003). VPI-2 is a 57.3 kb island integrated at *tRNA-Ser* and encodes a neuraminidase important for converting higher-order sialogangliosides to GM1 gangliosides, the receptor for cholera toxin (Galen *et al*., 1992). Moreover, VPI-1 encodes for the toxin-coregulated pilus (TCP), an essential intestinal colonization factor, as well as the accessory colonization factor (ACF), and virulence regulators ToxT and TcpPH (Everiss *et al*., 1994; Murphy and Boyd, 2008). Non-O1/O139 *V. cholerae* strains are considered to be the major source of laterally acquired DNA for O1/O139 strains (Meibom *et al*., 2005) thus, a better understanding of the diverse genetic elements present in the *V. cholerae* species is important for predicting and mitigating the emergence of new pandemic strains.

In bacteria, errors in DNA can occur as part of normal DNA replication or can be induced by external stimuli (e.g. UV irradiation) (Janion, 2008). There are several genetic systems involved in error-free DNA repair including base excision repair (BER), nucleotide excision repair (NER), recombinational DNA repair and mismatch repair (MMR) (Rattray and Strathern, 2003; Janion, 2008; Polosina and Cupples, 2010; Lenhart *et al*., 2012). However, if DNA damage is extensive the mutagenic phase of the SOS response is triggered (Goodman, 2002). This response is mediated by DNA polymerases that replicate past template lesions in a process called translesion DNA synthesis (TLS) that is inherently error-prone (Goodman, 2002). For example, DNA polymerase V, encoded by the *umuDC* operon (Patel *et al*., 2010). The SOS induction of error-prone polymerases is considered a final response where although induced mutation(s) may be deleterious to the host cell, this is balanced against the need for rapid DNA repair (Goodman, 2002). An alternative view for the function of error-prone polymerases is that they act to generate genetic diversity that may have a role in environments where the host is maladapted by providing a bank of pre-existing genetic diversity within that population, some of which may confer a positive selective advantage. To support this second view, transcription of error-prone polymerases has been observed in the absence of SOS inducing DNA damage (Yeiser *et al*., 2002). Furthermore, error-prone polymerase mutants are less competitive than the parent cells during starvation (McKenzie *et al*., 2000; Yeiser *et al*., 2002; Tark *et al*., 2005), and some antibiotics (e.g. quinolones) induce the SOS mutagenic response increasing the frequency of resistant mutants (Piddock and Wise, 1987; Ysern *et al*., 1990).

In this study we report a novel GI inserted into *recA* of *V. cholerae* non-O1/O139 strain S24 isolated from an estuarine river in Sydney, Australia. This strain lacks the major virulence factors: cholera toxin and the toxin-coregulated pilus, thus is not capable of causing cholera. The GI carries (i) a *recA* gene phylogenetically distant from the disrupted host *recA*, designated *recA*_RME_; (ii) a *umuDC* operon, designated *umuDC*_RME_, encoding DNA polymerase V; and (iii) genes encoding hypothetical proteins, proteins with DNA processing domains including a MutL domain involved in MMR, and proteins involved in site-specific recombination. The GI can excise as a closed circle and preferentially inserts into a specific site within *recA*. We also show that *recA*_RME_ is functional and provides protection from UV irradiation, a common source of DNA damage encountered in the shallow waters of marine and estuarine environments. Furthermore, the GI provides protection from the antibiotics bleomycin and ciprofloxacin. Acquisition of this GI by O1/O139 toxigenic *V. cholerae* would not only enhance survival of this pathogen in the natural environment but may also provide enhanced protection from DNA targeting antibiotics such as ciprofloxacin.

## Results and discussion

### Identification of a novel genomic island in *V. cholerae* S24 containing *recA*

**Fig 1:**
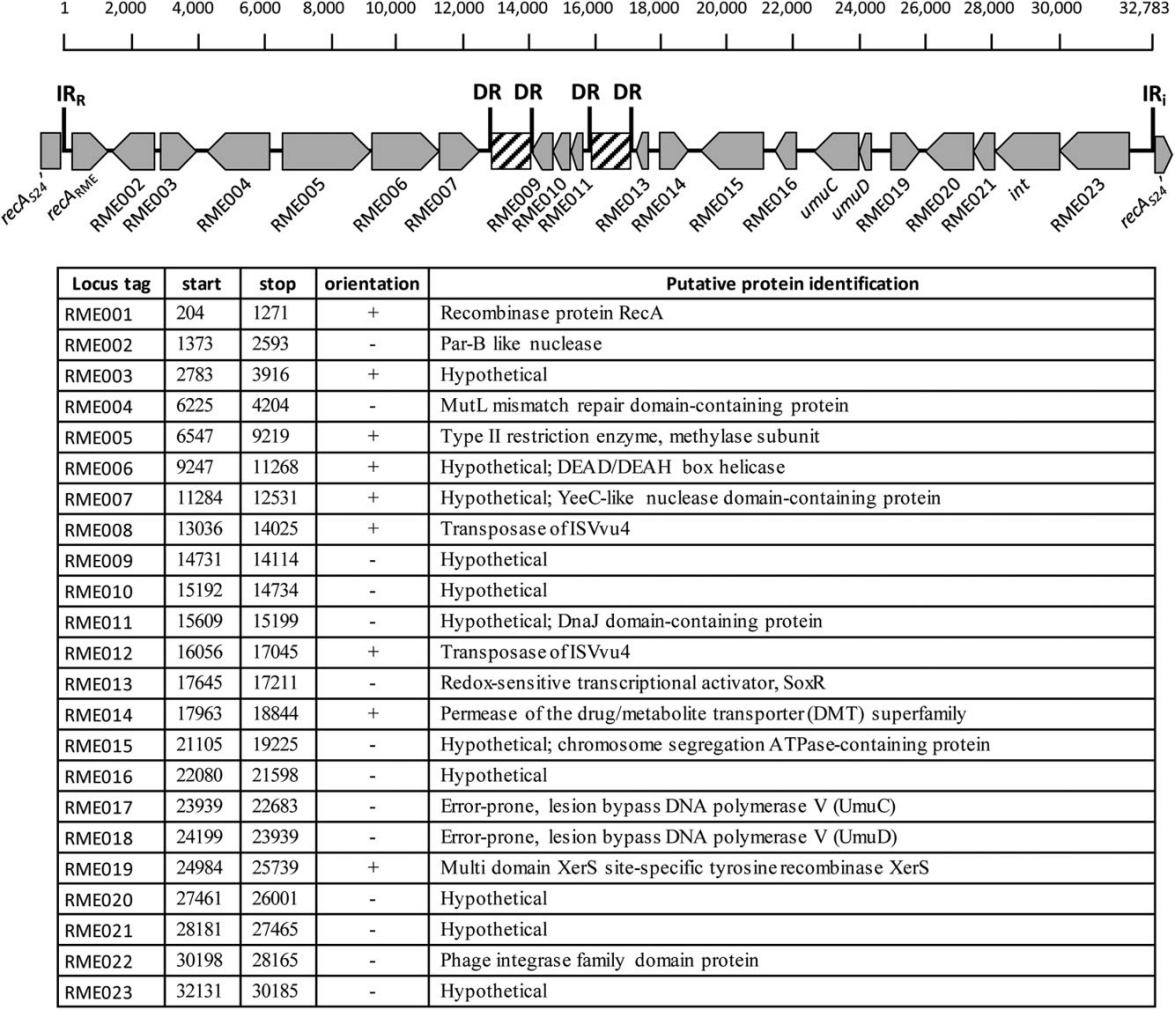
Genetic structure and gene content of the *recA* genomic island. The RME contains 9 bp inverted repeats at each end (IR_R_ and IR_i_) and 23 ORFs inclusive of the transposase genes from the ISVuv4 elements (striped boxes). The ISVuv4 elements are abutted by 7 bp direct repeats (DR) indicating insertion by transposition. RME contains multiple genes in DNA repair including a full copy of *recA* (RME001), the *umuDC* operon (RME017 and RME018) encoding the two subunits of DNA polymerase V and a gene encoding a protein with a MutL mismatch repair domain (RME004).

S24 is an environmental, non-O1/O139 *V. cholerae* strain isolated from Georges River in Sydney, Australia, as described in a previous study (Islam *et al*., 2013). It was noted during multilocus sequence analysis of housekeeping genes *adk*, *gyrB*, *mdh* and *recA* using primers designed to amplify the *V. cholerae* S24 *recA,* (designated here *recA*_S24_), that a product of ∼ 1.5 kb was identified instead of the expected ∼ 850 bp. When the *V. cholerae* S24 draft genome sequence (to be released at a later date) was interrogated, it was noted that *recA*_S24_ was present on two separate contigs. PCR, using primers designed to sequence within these contigs, was used to close this region of the genome (described in *Experimental procedures*) resulting in a final contig of 262,869 bp. Within this contig and disrupting *recA*_S24_ at 494 bp into the 1065 bp gene was a GI of 32,787 bp we have designated *recA* mobile element (RME). Consistent with RME being a mobile genetic element, the GC content is 41.3% compared with the genome average of 47.2%, it encodes mobility functions (see below) and is bordered by 9 bp inverted repeats, designated IR_R_ (for ***r****ecA* end) and IR_i_ (for **i**ntegrase end) (Fig. 1). Bioinformatic analysis of the GI identified 23 coding sequences (CDSs) (Fig. 1) including a complete copy of *recA,* designated *recA*_RME_ at the IR_R_ end and a phage integrase at the IR_i_ end. To our knowledge, this is the first mobile genetic element associated with the lateral movement of the critical gene, *recA*.

A number of other genes similar to those known to be involved in DNA processing are also found in RME including *umuDC* encoding the error-prone DNA polymerase V and a gene encoding a protein with a partial domain found in MutL (COG0323; Fig. 1), a component of the MMR pathway (Polosina and Cupples, 2010). A number other genes on RME, homologous to those involved in DNA processes include those encoding a ParB*-*like nuclease (91% identity to *Vibrio alginolyticus* 12G01; WP_005381205.1), a redox sensitive transcriptional activator with a SoxR*-*domain (96% identity to *Vibrio* sp. 712i1; WP_017634100.1), a type II restriction enzyme containing a methylase subunit (77% identity to *Vibrio splendidus*; WP_017082665.1) and a helicase (90% identity to *Vibrio brasiliensis* LMG 20546; WP_006880978.1).

Two insertion sequence (ISVvu4) elements were identified at positions 12,877 – 14,083 and 15,897 – 17,103 (striped boxes in Fig. 1) of RME. In both instances, 7 bp direct repeats (DR) were evident bordering the ISVvu4 elements indicating insertion by transposition. The DR for each ISVvu4 element is different, indicating independent insertion events. *In silico* removal of the ISVvu4 elements from the sequence did not restore any CDSs indicating that their insertion had not led to gene disruption. As expected, the promoter regions of both *recA*_RME_ and the *umuDC*_RME_ operon have the characteristic LexA binding sequence of CTGT-(AT)_4_-ACAG indicating control by the SOS response (Wertman and Mount, 1985; Sanchez-Alberola *et al*., 2012). Present on RME are also genes putatively involved in mobilization/integration such as a phage integrase (RME022) and a site-specific recombinase XerS (RME019) (Fig. 1).

**Fig 2:**
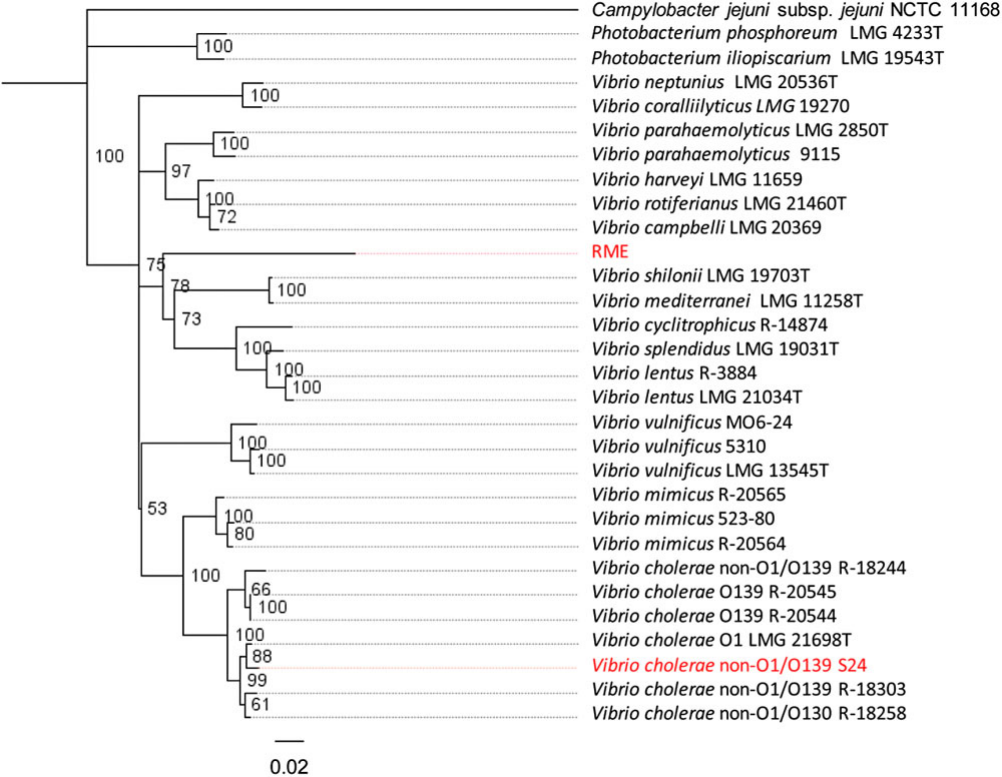
Phylogenetic analysis of *recA*_S__24_ and *recA*_RME_ (RME highlighted in red) *recA*_S__24_ (also highlighted in red) groups with *V. cholerae* strains whereas, *recA*_RME_ groups with *recA* from other *Vibrio* species indicating that *recA*_RME_ was mobilized from another member of the *Vibrio* genus.

Phylogenetic analysis of *recA* sequences from the *Vibrionaceae* determined that *recA*_S24_ is characteristic of *recA* genes found within the *V. cholerae* clade, whereas *recA*_RME_ is not. It does, however, group with other more distantly related *recA* genes found in other members of the *Vibrio* genus (Fig. 2). This is consistent with *recA*_RME_ having been acquired by LGT. *recA* is an excellent phylogenetic marker for resolving relationships within the *Vibrionaceae* family (Stine *et al*., 2000; Thompson *et al*., 2004). Although the acquisition of a divergent *recA* in *V. cholerae* S24 is easily evident, this data reminds us that LGT of critical housekeeping genes like *recA* can and does occur. Less evident would be LGT of *recA* between closely related strains within the *V. cholerae* species confounding phylogenetic trees using a single marker (Bapteste *et al*., 2004; Creevey *et al*., 2004).

### The *recA* mobile element excises as a closed circle and targets a specific site in *recA*

**Fig 3:**
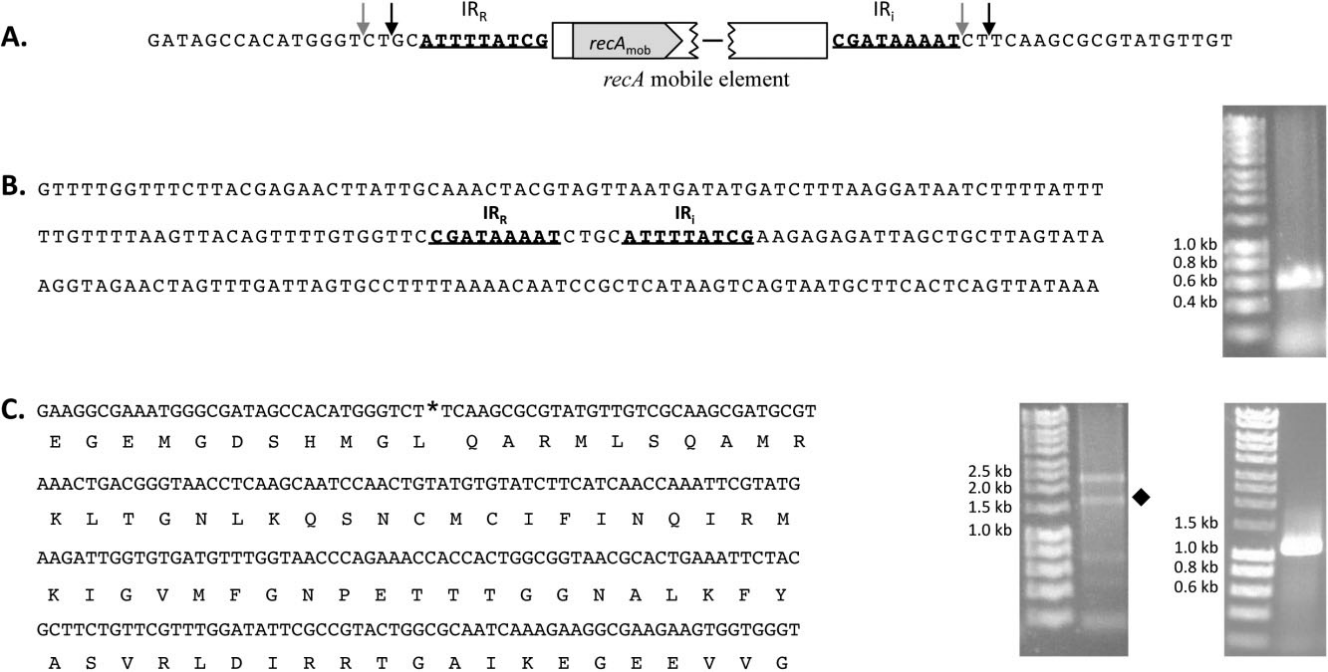
A. Sequence abutting insertion of the *recA* genomic island in *V. cholerae* S24. The black and grey arrows demarcate the possible excision points for the RME.B. Sequence and gel image of the product derived from inverse PCR of the excised RME. The sequence shows that excision does not precisely occur at IR_R_ and IR_i_ and either occurs by two possible methods shown in Fig. 3A (see text for more details).C. Sequence of the ‘empty’ *recA*_S__24_ insertion site and translated peptide sequence shows excision restores an uninterrupted *recA*_S__24_. The asterisk marks the point of RME insertion. Amplification of the ‘empty’ *recA*_S__24_ site gave a faint product (marked by diamond in left gel image). This was excised, purified and a nested PCR (right gel image) was conducted to generate sufficient product for sequencing.

Many GIs are known to excise from their location in the chromosome (Boyd *et al*., 2008). Analysis of RME suggested that it integrated into *recA*_S24_ using site-specific recombination. In site-specific recombination, a DNA recombinase recognizes specific sequences (usually inverted sequences) allowing for DNA breakage and joining reactions that result in integration or excision of the element (Hallet and Sherratt, 1997). Exact excision of RME at IR_R_ and IR_i_ was predicted to leave behind a 4 bp scar introducing a frame shift in *recA*_S24_ (Fig. 3A). To determine whether RME excision would leave behind an excision scar, an inverse PCR was conducted using primers reading out from the IR_R_ and IR_i_ ends (primers RME-F/RME-R in Table 1). A product of ∼ 560 bp was amplified (see gel image in Fig. 3B) and sequenced. Analysis of the sequence showed that excision occurred in one of two possible ways (Fig. 3B): (1) Excision occurred at 2 bp on either side of the IR_R_ and IR_i_ ends (black arrows in Fig. 3A) and/or (2) precise excision occurred at the end of IR_i_ and at 4 bp before the end of IR_R_ (grey arrows in Fig. 3A). Either way, excision was predicted to restore an uninterrupted and therefore functional copy of *recA*_S24_ in the chromosome, consistent with site-specific recombination. This was confirmed by amplification of an intact ‘empty’ insertion site using primers S24-cinA-F/S24-recX-R and excising the predicted ∼ 1.6 kb fragment (marked with a diamond in Fig. 3C). A nested PCR was then performed on the purified excised fragment using primers EcoRI-recA-F/EcoRI-recA-R (see gel image in Fig. 3C) and the product sequenced (Fig. 3C).

**Table 1:** Primers used in this study

Primer	Sequence (5′-3′)	Target	Source
RME-R	GACGAGTCCAGCTCATGACA	integrase end of *recA* genomic island	This study
RME-F	GCTGCTAACGCTTTCTGCTT	*recA* end of *recA* genomic island	This study
S24-ctg675-F	CGGTTAGGAGGGGCTTTTAG	3′ end of contig 675	This study
S24-ctg708-R	TATCGGCTGTGGTTGTTTGA	5′ end of contig 675	This study
S24-ctg367-F	TAGCTAGAGCATTTGTCATAAGAAAAAGTAAG	3′ end of contog 675	This study
S24-ctg367-R	ACTGGCAGCAGAAGAAGCAT	5′ end contig 708	This study
S24-cinA-F	CAAGGTTGGCTCAAAGTG	*cinA* in *V. cholerae* S24	This study
S24-recX-R	GGCATCACTCAAATACCCTA	*recX* in *V. cholerae* S24	This study
S24-recA-F	CTGGAAATTTGTGATGCATT	*recA* in *V. cholerae* S24	This study
EcoRI-recA-F^a^	TTTT**GAATTC**TGGACGAGAATAAACAGAAGG	*recA* in *V. cholerae* S22 & S24	This study
EcoRI-recA-R^a^	TTTT**GAATTC**AAACTCTTCTGGCACCGC	*recA* in *V. cholerae* S22 & S24	This study
EcoRI-Ori700-R^a^	TTTT**GAATTC**CGCGCTATCGCTTGTCG	*ori_pB1067_* of pOriVn_700_	This study
EcoRI-OriR6K-F^a^	TTTT**GAATTC**GTGTTCCTGTGTCACTCAAAATTG	*ori6k*	This study
Ori700-F	CCCTATTCCTCTTTAGTCCTGC	*ori_pB1067_* of pOriVn_700_	This study
Ori6K-R	TAACGCACTGAGAAGCCC	*ori6k*	This study
S24-phage-Int-F	GCCAAGATATGGCAGGAAAA	Integrase in *recA* genomic island	This study
S24-phage-Int-R	GGACGCTACCCAGTGAATGT	Integrase in *recA* genomic island	This study
recA-F	TGGACGAGAATAAACAGAAGGC	*recA*	(Boucher *et al*., 2011)
recA-R	CCGTTATAGCTGTACCAAGCGCCC	*recA*	(Boucher *et al*., 2011)
pCC2FOS-FP	GTACAACGACACCTAGAC	pCC2FOS sequencing primers (F)	Epicentre Biotechnologies
pCC2FOS-RP	CAGGAAACAGCCTAGGAA	pCC2FOS sequencing primers (R)	Epicentre Biotechnologies
recA-Tn5-F	CGCTCATAAGTCAGTAATGCTTCA	*recA* on genomic island. Used to screen for Tn*5* insertion.	This study
umuC-Tn5-F	GATGTATGGCTGAATCGACCA	*umuC* on genomic island. Used to screen for Tn*5* insertion.	This study
KAN-2 FP-1	ACCTACAACAAAGCTCTCATCAACC	Forward primer inside Tn5 used to screen for Tn5 insertion.	Epicentre Biotechnologies
KAN-2 RP-1	GCAATGTAACATCAGAGATTTTGAG	Reverse primer inside Tn5 used to screen for Tn5 insertion.	Epicentre Biotechnologies

a Bold and underlined sequence shows the *Eco*RI restriction site.

**Fig 4:**
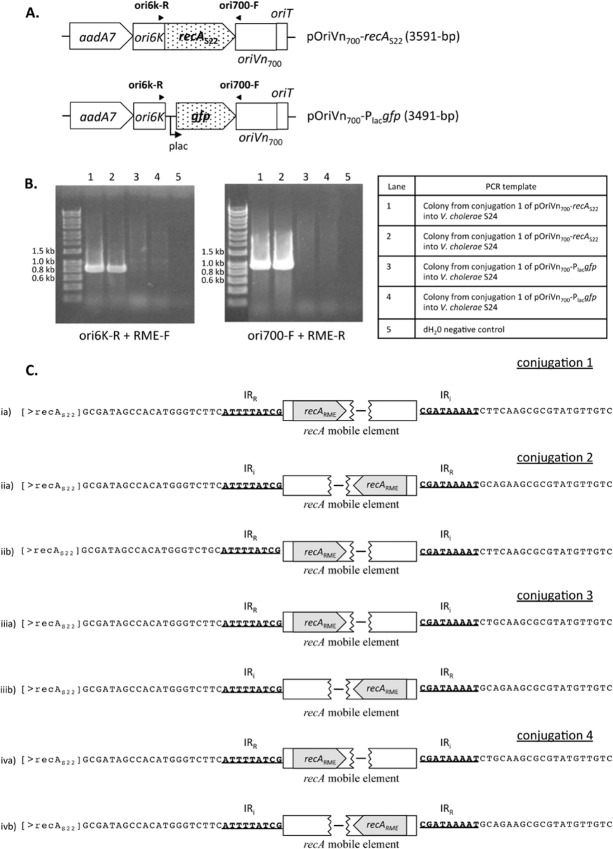
Translocation of the *recA* genomic island from the genome of *V. cholerae* S24 into a replicating vector containing *recA*_S__22_.A. Genetic structure of the replicating vectors pOriVn_700_-*recA*_S__22_ and pOriVn_700_-P_lac_*gfp* and the placement of primers ori6k-R and ori700-F used for amplifying the boundaries of the translocated genomic island are shown.B. Representative gel showing amplification using vector specific and RME specific primers from colonies derived from conjugation of pOriVn_700_-*recA*_S__22_ (lanes 1 and 2) and pOriVn_700_-P_lac_*gfp* (lanes 3 and 4) into *V. cholerae* S24. Lane 5 shows negative dH_2_O control.C. Sequence of products derived using vector-specific and RME-specific primers from PCR of *V. cholerae* S24 transconjugates from four independent conjugations. Each transconjugant is denoted by i, ii, iii and iv. In most instances (iib, iiib and ivb), the same transconjugate showed insertion of RME in both orientations relative to *recA*_S__22_. The sequences indicate specific insertion of RME into the same site of *recA*_S__22_ (the equivalent insertion site in *recA*_S__24_).

To determine whether the RME was capable of translocating from the genome of *V. cholerae* S24 into a new location, a vector (pOriVn_700_-*recA*_S22_; see Fig. 4A) containing the *recA* gene from a closely related strain of *V. cholerae* S24, strain S22 (Islam *et al*., 2013), was introduced into *V. cholerae* S24 by conjugation. Here, the RME is expected to excise from the genome of *V. cholerae* S24 and insert into *recA*_S22_ present on pOriVn_700_-*recA*_S22_. A control vector substituting *recA*_S22_ with *gfp* (pOriVn_700_-P_lac_*gfp*; see Fig. 4A) was also introduced into *V. cholerae* S24 by conjugation as a control. Primers (ori6k-R and ori700-F; see Fig. 4A) targeting the vector backbone and the ends of the RME were used in a PCR reaction to determine whether the RME had mobilized into either pOriVn_700_-*recA*_S22_ or pOriVn_700_-P_lac_*gfp*. In four independent experiments where pOriVn_700_-P_lac_*gfp* was successfully introduced into *V. cholerae* S24 by conjugation, a product was never detected in the transconjugates (see representative gel in Fig. 4B). However, when pOriVn_700_-*recA*_S22_ was successfully introduced into *V. cholerae* S24, products were amplified (see representative gel in Fig. 4B) demonstrating insertion of the RME in the equivalent DNA site of *recA*_S22_ and in both orientations, with respect to *recA*_S22_. Sequence of the PCR products are shown in Fig. 4C, demonstrating successful insertion of RME into the equivalent *recA*_S24_ insertion site into *recA*_S22_ in pOriVn_700_-*recA*_S22_.

It should be noted that homologous recombination between *recA*_S22_ in pOriVn_700_-*recA*_S22_ and *recA*_S24_ in the *V. cholerae* S24 genome could result in merodiploids that generate the same amplicons as those for RME inserted in the orientation (relative to *recA*_S24_) found in *V. cholerae* S24 (see Supporting Information Fig. S1 on expected merodiploids). However, in the immediate 2 bp of the IR_R_ end for three of the transconjugates (Fig. 4C; ia, iiia and iva), there is a G to T substitution and at the immediate 3 bp of the IRi end, two transconjugants (Fig. 4C; iiia and iva) showed a T to G substitution. Since the *recA*_S22_ sequence is identical to *recA*_S24_ around the insertion point, homologous recombination should result in identical sequences immediately surrounding the RME. Furthermore, the RME was also found in both orientations, with respect to *recA*_S22_ (Fig. 4C). Consequently, homologous recombination is unable to explain these results.

These data show that RME is capable of mobilization and preferentially targets a specific site within *recA*. By carrying its own functional copy of *recA,* the GI does not affect any of the vital cell pathways associated with disruption of this gene during integration. Furthermore, specific targeting of *recA* may be necessary to ensure successful maintenance and dissemination of the GI. Since RecA does not function as a monomer but polymerizes to form a filament structure (Yu *et al*., 2004), disruption of the indigenous *recA* prevents a situation where two divergent RecA proteins might negatively interact resulting in reduced cell fitness.

### The *recA* mobile element provides *E. coli* protection from UV irradiation

**Fig 5:**
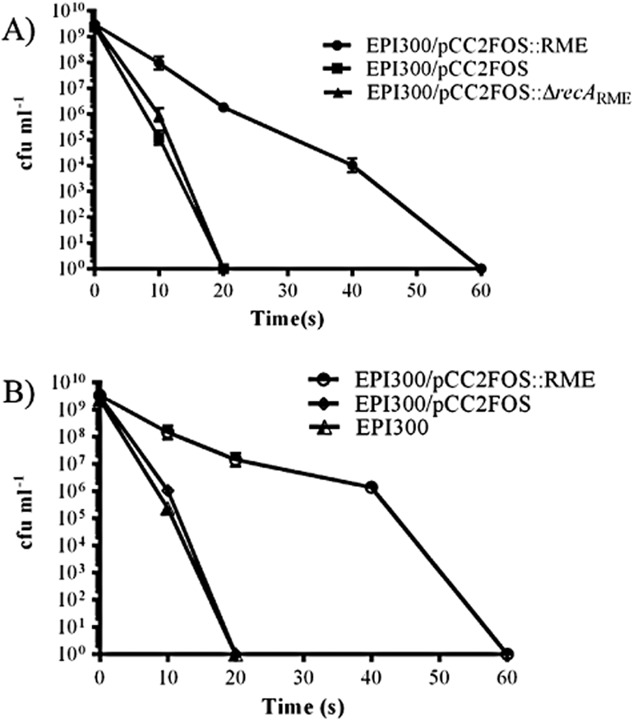
Survival of *E. coli* carrying the *recA* genomic island and control strains when exposed to UV-C stress. Time points are given at 0, 10, 20, 40 and 60 s. UV-C exposure was set to 0.8 mJ cm^−2^.

The presence of multiple genes involved in DNA repair prompted us to look at whether the GI could protect against a common DNA-damaging process faced by *V. cholerae* – UV irradiation. To investigate if *recA*_RME_ has a role in protecting the cell from DNA damage, the RME was cloned into a fosmid and used to transform *recA^-^ E. coli* strain EPI300. The resultant transformant was subjected to UV-C irradiation. Fig. 5A shows that the presence of the RME element conferred enhanced bacterial cell survival when exposed to 0.8 mJ cm^−2^ of UV-C. From Fig. 5A and B it can be seen that EPI300 and EPI300 transformed by vector only controls are completely killed by exposure to 20 s 0.8 mJ cm^−2^ of UV-C. However, EPI300 transformants containing the RME survive for up to 60 s of UV-C exposure and show a 100-fold increase in survival at 10 s and up to 10,000,000-fold higher survival after 20 s UV-C exposure. Fig. 5A shows that when *recA*_RME_ is insertionally inactivated, the level of cell survivability decreases to a level comparable with the vector-only control (Fig. 5B). This demonstrates that *recA*_RME_ is functional and is the gene mainly responsible for the protection provided by the presence of the RME. An interesting future question would be whether *recA*_RME_ is more efficient in DNA repair than the host *recA* (i.e. *recA*_S24_). There is precedent for such an idea, in a strain of *Clostridium difficile,* a 4.2 kb insert disrupts a gene encoding a thymidylate synthetase (involved in DNA synthesis and repair) but contains a more functionally active version of the disrupted gene (Knetsch *et al*., 2011).

### The *recA* mobile element provides *E. coli* with increased protection against antibiotics

Since RME has multiple genes involved in DNA repair, we tested whether RME provided enhanced protection against three DNA-targeting antibiotics: nalidixic acid, ciprofloxacin and bleomycin. Minimum inhibitory concentrations (MICs) were determined using nalidixic acid, ciprofloxacin and bleomycin (Table 2). Interestingly, the MIC of the first-generation quinolone, nalidixic acid, did not vary between any of the tested strains, including the RME (data not shown). However, when the strains given in Table 2 were tested using ciprofloxacin, a second-generation quinolone, a fourfold increase in MIC was observed for strains containing the RME. In the case of ciprofloxacin, it is apparent that *recA*_RME_ is responsible for the increased resistance. When *recA*_RME_ is insertionaslly inactivated from the genomic island, the MIC drops to a level equivalent to that seen for the *E. coli* strain EPI300. The importance of *recA* in protection against ciprofloxacin and other antibiotics has previously been documented [e.g. in *Acinetobacter baumannii* (Aranda *et al*., 2011)]. RME also provided protection from bleomycin in *E. coli* EPI300. However, in contrast to ciprofloxacin, when *recA*_RME_ and *umuC* from the RME (designated *umuC*_RME_) are both inactivated independently, the MIC against bleomycin is the same as *E. coli* EPI300, indicating that RecA_RME_ activation of the DNA polymerase V subunit UmuD to UmuD’ encoded on the GI is responsible for protections against bleomycin (Patel *et al*., 2010). Since *umuDC*_RME_ was able to provide protection from bleomycin in a genetic background that already contains *umuDC*, it is hypothesized that in the *V. cholerae* species, where *umuDC* is only sporadically found, this element may provide increased protection from DNA damage compared with what was observed here in *E. coli* EPI300.

**Table 2:** Minimal inhibitory concentration (MICs^a^)

Strain	Ciprofloxacin	Bleomycin
EPI300	0.015625	8
EPI300/pCC2FOS	0.015625	8
EPI300/pCC2FOS::RME	0.0625	16
EPI300/pCC2FOS::RMEΔ*umuC*_RME_	0.0625	8
EPI300/pCC2FOS::RMEΔ *recA*_RME_	0.015625	8

a MIC given as μg ml^−1^.

Apart from DNA repair, *recA* and DNA polymerase V (encoded by *umuDC*) are known to increase spontaneous mutation frequencies resulting in the emergence of antibiotic resistance mutants (Thi *et al*., 2011). Spontaneous mutation in *V. cholerae* is well documented to cause resistance to a variety of antibiotics (Goss *et al*., 1965; Gellert *et al*., 1977; Sugino *et al*., 1977; Allen *et al*., 1979; Kitaoka *et al*., 2011). Here we chose to examine the mutation frequency of two antibiotics, rifampicin which acts on protein synthesis and nalidixic acid which targets DNA replication by inhibiting the A subunit of DNA gyrase. Mutation frequencies after 24 and 48 h showed no differences between *E. coli* containing RME and the controls on 100 μg ml^−1^ rifampicin (data not shown). This may be because of rifampicin acting on protein synthesis and therefore not inducing the SOS response which induces transcription of *umuDC*. However, when the experiments were repeated with 50 μg ml^−1^ nalidixic acid, *E. coli* EPI300 and the vector-only control consistently did not produce any spontaneous mutants, while *E. coli* EPI300/pCC2FOS::RME showed an increased mutation frequency after both 24 and 48 h incubation in the presence of nalidixic acid (Table 3). *E. coli* EPI300/pCC2FOS::RMEΔ*umuC*_RME_ generally had the same mutation frequency as the complete RME. However, experiment 3 (Table 3) showed that this strain produced no mutants after 24 and 48 h. It can be concluded from these experiments that *E. coli* EPI300 with the complete RME provides an adaptive advantage by increasing the mutation rate resulting in subsequent resistance to nalidixic acid, but this effect could not be wholly attributed to the activity of *umuC*_RME_. One possible explanation is the activation of the indigenous *E. coli* UmuD by RecA provided by the GI. However, it cannot be excluded that other genes on the RME are elevating the spontaneous mutation rate. Nevertheless, *umuDC*-like operons are commonly associated with mobile genetic elements (Permina *et al*., 2002; Tark *et al*., 2005; Hare *et al*., 2012) and do provide a general adaptive advantage to hosts that house them (Yeiser *et al*., 2002; Tark *et al*., 2005). Although we failed in our attempts to transfer RME into seven non-O1/O139 *V. cholerae* strains from Sydney using chitin transformation, this element is likely to do the same in a *V. cholerae* genetic background.

**Table 3:** Nalidixic acid^a^ mutation frequencies

Experiment 1		

Strain	Mutation frequency 24 h	Mutation frequency 48 h
EPI300	< 1.1 × 10^-11^	< 1.1 × 10^-11^
EPI300/pCC2FOS	< 1.7 × 10^-11^	< 1.7 × 10^-11^
EPI300/pCC2FOS::RME	1.4 × 10^-8^ (4)	1.5 × 10^-8^ (4)
EPI300/pCC2FOS::RMEΔ*umuC*_RME_	1.6 × 10^-9^ (3)	2.1 × 10^-9^ (4)

Experiment 2		

Strain	Mutation frequency 24 h	Mutation frequency 48 h
EPI300	< 1.1 × 10^-11^	< 1.1 × 10^-11^
EPI300/pCC2FOS	< 1.7 × 10^-11^	< 1.7 × 10^-11^
EPI300/pCC2FOS::RME	8.3 × 10^-10^ (1)	1.4 × 10^-9^ (3)
EPI300/pCC2FOS::RMEΔ*umuC*_RME_	2.8 × 10^-9^ (6)	4.4 × 10^-9^ (7)

Experiment 3		

Strain	Mutation frequency 24 h	Mutation frequency 48 h
EPI300	< 1.1 × 10^-11^	< 1.1 × 10^-11^
EPI300/pCC2FOS	< 1.3 × 10^-11^	< 1.3 × 10^-11^
EPI300/pCC2FOS::RME	4.8 × 10^-10^ (1)	1.7 × 10^-9^ (4)
EPI300/pCC2FOS::RMEΔ*umuC*_RME_	< 9.1 × 10^-12^	< 9.1 × 10^-12^

a Concentration of nalidixic acid = 50 μg ml^−1^.

‘<’ indicates that zero colonies appeared in all 10 replicates (see *Experimental procedures*).

Numbers in brackets indicates the number of replicates in which one or more colonies appeared.

To conclude, this study reports a novel GI in *V. cholerae* that contains genes involved in multiple DNA repair pathways, including the critical housekeeping gene *recA* and genes encoding DNA polymerase V which in this study, we show to be functional. The presence of other DNA processing genes may provide *V. cholerae* with alternative DNA repair pathways. Since this element can excise from its chromosomal location, it has the potential to mobilize into other strains, such as cholera toxin-producing O1/O139 pandemic strains. Such mobilization could have implications for increased environmental survival or resistance to certain antibiotics.

## Experimental procedures

### Bacterial strains, plasmids and growth conditions

All strains and plasmids used are shown in Table 4. *V. cholerae* strain S24 was collected from Georges River in the greater Sydney (Australia) urban area as previously described (Islam *et al*., 2013). All *E. coli* and *V. cholerae* strains were routinely grown on Luria–Bertani (LB) broth at 37°C under aerobic conditions. For *E. coli* WM3064, diaminopimelic acid (DAP) was added to a final concentration of 0.3 mM. Spectinomycin was used for *E. coli* and *V. cholerae* at 50 μg ml^−1^ and 125 μg ml^−1^ respectively. Chloramphenicol was used at 12.5 μg ml^−1^.

**Table 4:** List of strains and plasmids

Strain or plasmid	Relevant genotype^a^	Reference or source
*V*. *cholerae*		
S24	Wild-type (non-O1/O139)	This study
S22	Wild-type (non-O1/O139)	(Islam *et al*., 2013)
*E. coli*		
DH5αλpir	*endA1 glnV*44 *thi-1 recA1 relA1 gyrA96 deoR nupG* Φ80d*lacZ*ΔM15 Δ(*lacZYA-argF*)U169 *hsdR17 λpir*	(Demarre *et al*., 2005)
WM3064	Donor strain for conjugation: *thrB1*004 *pro thi rpsL hsdS lacZ*Δ*M15* RP4-1360 Δ(*araBAD)567* Δ*dapA*1341::[*erm pir*], Sm^R^	(Saltikov and Newman, 2003)
EPI300™-T1^R^	[F^-^ mcrA Δ(*mrr-hsd*RMS-*mcr*BC) ϕ80d*lacZ*ΔM15 Δ*lacX*74 *rec*A1 *end*A1 *ara*D139 Δ(*ara, leu*)7697 *gal*U *gal*K λ^-^ *rps*L *nup*G *trf*A *ton*A *dhfr*], Sm^R^, Tp^R^	Epicentre Biotechnologies
Plasmids/fosmids		
pCC2FOS	Cloning vector, Cm^R^	
pCC2FOS-RME	pCC2FOS vector containing 32 kb insert from *V. cholerae* S24. The insert contains the *recA* GI and surrounding sequence, Cm^R^	This study
pCC2FOS::RMEΔ*recA*_RME_	pCC2FOS vector containing 32 kb insert from *V. cholerae* S24. The insert contains the *recA* GI and surrounding sequence and has *recA* on the GI insertionally inactivated by Tn5, Km^R^, Cm^R^	This study
pCC2FOS::RMEΔ*umuC*_RME_	pCC2FOS vector containing 32 kb insert from *V. cholerae* S24. The insert contains the *recA* GI and surrounding sequence and has *umuC* present on the GI insertionally inactivated by Tn5, Km^R^, Cm^R^	This study
pOriVn_700_	Low copy mobilizable vector containing *ori_pB1067_* (vibrio specific) and *ori6K*, Sp^R^	(Le Roux *et al*., 2011)
pOriVn_700_-*recA*_S22_	pOriVn_700_ with *recA* from *V. cholerae* S22 in between *ori_pB1067_* and *ori6K.* The *recA* gene is reading toward *ori_pB1067_*, Sp^R^	This study
pOriVn_700_-P_lac_*gfp*	pOriVn_700_ with P_lac_*gfp* cloned in between *ori_pB1067_* and *ori6K,* Sp^R^	(Le Roux *et al*., 2011)

a Tc^R^, tetracycline resistance; Sm^R^, streptomycin resistance; Sp^R^, spectinomycin resistance; Cm^R^, chloramphenicol resistance, Km^R^, kanamycin resistance.

### Whole genome sequencing, PCR, DNA extraction and sequencing methods

DNA was extracted using the Wizard genomic DNA purification kit (Promega). Plasmid and PCR/gel extractions were done using PureYield Plasmid Miniprep and Wizard SV Gel and PCR clean-up systems respectively (Promega). Purified DNA from *V. cholerae* S24 was sequenced at the Wellcome Trust Sanger Institute using Illumina-based technology.

All primers used in this study are shown in Table 1. Standard PCR was performed using the PCR master mix (Promega) containing 25 units ml^−1^ of *Taq* DNA polymerase, 800 μM dNTPs and 1.5 mM MgCl_2_. Primers were used at a final concentration of 0.5 μM each. All PCRs were performed with 30 cycles of denaturation at 94°C for 30 s, the appropriate annealing temperature for 30 s and an extension of 72°C (1 min kb^−1^) and sequencing performed at Macrogen. From whole genome sequencing (Wellcome Trust Sanger Institute) it became evident that the host *recA* had been disrupted and was present on two separate contigs. The two contigs (contigs 675 and 708) were pieced together by PCR and joined to an intervening third contig to produce a contig of 262,869 bp (contig 367) using primers described in Table 1. The accession number for RME is KJ123688.

### Cloning of RME and transposon mutagenesis of *recA* genomic island

To clone the *recA* genomic island (RME) from *V. cholerae* strain S24, genomic DNA was digested with *Nae*I and a library constructed using the CopyControl Fosmid Library Production Kit (Epicentre). *Nae*I digestion of *V. cholerae* strain S24 genomic DNA creates a fragment of 38, 913 bp containing the entire 32, 787 bp RME. The library was screened for a fosmid clone containing the 38, 913-bp *Nae*I fragment using primers targeting the phage integrase in the RME (Table 1). A positive clone designated pCC2FOS-RME was confirmed by sequencing the ends of the cloned insert using the pCC2FOS vector primers FP and RP (Table 1). To create the pCC2FOS no insert control, linearized and dephosphorylated pCC2FOS (Epicentre) was treated with T4 polynucleotide kinase and circularized by ligation. A mutant library of pCC2FOS-RME was constructed using the EZ-Tn5 Kan-2 Insertion Kit (Epicentre Biotechnologies) according to manufacturer instructions. Mutants containing knockouts of individual genes present on the genomic island were screened by PCR using primers reading out from EZ-Tn5 Kan-2 and a primer targeting the gene of interest (Table 1).

### Phylogenetic analysis

Phylogenetic analysis of *recA*_S24_ and *recA*_RME_ was done using bioinformatics program Geneious version 6.1.6 and FigTree version 1.4.0. Phylogenetic tree parameters were taken from (Thompson *et al*., 2004). Distance estimations were obtained using the Jukes and Cantor model and tree built using the neighbour-joining method. Bootstrap percentages were calculated after 100 simulations. The *Campylobacter jejuni* subsp. *jejuni* NCTC 11168 *recA* sequence was used as an outgroup.

### *recA* targeting experiments

Vector pOriVn_700_ and *recA* from a strain of *V. cholerae* S22 that is closely related to *V. cholerae* S24 were amplified using primer pairs EcoRI-Ori700-R/EcoRI-Ori6K-F and EcoRI-recA-F/EcoRI-recA-R respectively (Table 1). Since the primers contained engineered *Eco*RI sites, the resulting amplicons of *recA*_S22_ and pOriVn_700_ were purified, digested with *Eco*R1 and then ligated together using T4 DNA ligase (Fermentas). The ligation mix was then transformed into *E. coli* DH5αλpir to produce pOriVn_700_-*recA*_S22_. The construct was then extracted and transformed into the conjugation donor strain *E. coli* WM3064.

Conjugations using pOriVn_700_-*rec*A_S22_ and pOriVn_700_-P_lac_*gfp* were performed by combining equal volumes of overnight cultures in LB from both donor and recipient strains. These were then centrifuged at 3000 × *g* and re-suspended in 50 μL of LB and spotted onto a 0.2 μM filter (Millipore) that had been placed on an LB agar plate containing 0.3 mM DAP. Donor and recipient cells were left to incubate for 4 h at 37°C and cells were then removed from the filter by vortexing. The re-suspended cells were then plated on LB + 125 μg ml^−1^ spectinomycin and incubated at 37°C overnight. One colony per mating was picked and appropriate junction PCR was conducted using primers in plasmid backbone (Table 1; Ori700-F/Ori6K-R) and primers reading out from RME (Table 1; RME-F/RME-R).

### UV stress experiments

UV stress experiments were adapted from Lin and Wang (2001). Strains were grown for 16–20 h at 37°C with shaking at 230 r.p.m. in 5 ml LB broth supplemented with appropriate antibiotic. Cells were centrifuged at 4000 × *g*, corrected for differences in optical density (OD) at 600 nm and re-suspended in equal volumes of M9 salts (Sambrook *et al*., 1989) supplemented with MgSO_4_.7H_2_O to a final concentration of 0.002 M. The entire cell suspension was placed in a clear bottom 10 cm plastic petri dish and subjected to 0.8 mJ cm^−2^ UV-C for 0, 10, 20, 40 and 60 s using an Amersham Life Science Ultraviolet Crosslinker. After each time interval, 150 ul aliquot was removed and placed in a 1.5 ml Eppendorf tube in the dark. The remaining liquid culture was thoroughly re-suspended using a pipette to avoid clumping of cells. After the final UV-C exposure time point, cells were diluted in M9 salts+ MgSO_4_.7H_2_O to 10^-6^ and enumerated by the drop plate method on LB agar. Plates were incubated in the dark to prevent photoreactivation at 37°C overnight and colony-forming units (CFUs) were calculated the following day.

### Minimum inhibitory concentration experiments and antibiotic mutation frequency experiments

MICs of nalidixic acid, ciprofloxacin and bleomycin were determined by broth microdilution using standard methods (Clinical and Laboratory Standards Institute, 2003) except that LB broth was used as the growth medium instead of Mueller Hinton. Each MIC was performed in triplicate. The mutation frequency experiment was designed using the guidelines described in (Pope *et al*., 2008). Specifically, mutation frequencies were determined using LB supplemented with 50 μg ml^−1^ nalidixic acid and 100 μg ml^−1^ rifampicin. Ten replicate overnight cultures for each strain were grown in 5 ml LB (chloramphenicol was added for those strains carrying pCC2FOS and derivatives). Each overnight culture was then diluted to ∼ 10^4^ CFU ml^−1^ with fresh LB5 (no chloramphenicol added) and 5 ml for each replicate was transferred into a 15 ml tube and incubated for 16–20 h at 37°C with shaking at 230 r.p.m. The following day, 200 μl from each tube was spread plated onto LB5 agar supplemented with the appropriate antibiotic (rifampicin or nalidixic acid) and incubated for 24 h and then 48 h at 37°C when colonies were counted. This was repeated in triplicate. In order to calculate total colony counts, cells were enumerated on LB5 agar with no antibiotic. Note that these experiments were performed in a Class II Biosafety Hood to avoid any contamination. Mutation frequencies were calculated as number of antibiotic-resistant CFUs/total number of CFUs after 24 and 48 h.

## Supporting information

**Fig. S1.** Possible insertion and homologous recombination events of the *recA* genomic island with pOriVn_700_-*recA*_S22_. Production of merodiploids because of homologous recombination between *recA*_S22_ and *recA*_S24_ could result in two genetic structures (crossover 1 and crossover 2). In both instances, PCR products could be generated using the vector-specific and RME-specific primers used to detect insertion of the RME into *recA_S22_* (broken lines) in the orientation found in the *V. cholerae* S24 genome (insertion 1). Insertion of the RME in the inverse orientation (insertion 2) would generate products using inverse primer pairs. The inverse insertion cannot be explained by homologous recombination and indicates an integration event.

**Appendix S1.** Genbank file of the *recA* mobile element (RME); Accession Number KJ123688.
