# Supplementary figures and images for: A genomic island integrated into recA of Vibrio cholerae contains a divergent recA and provides multi-pathway protection from DNA damage
Source: Environ Microbiol. 2014 Jun 26;17(4):1090–102. doi: 10.1111/1462-2920.12512 (PMC4405046; doi:10.1111/1462-2920.12512)

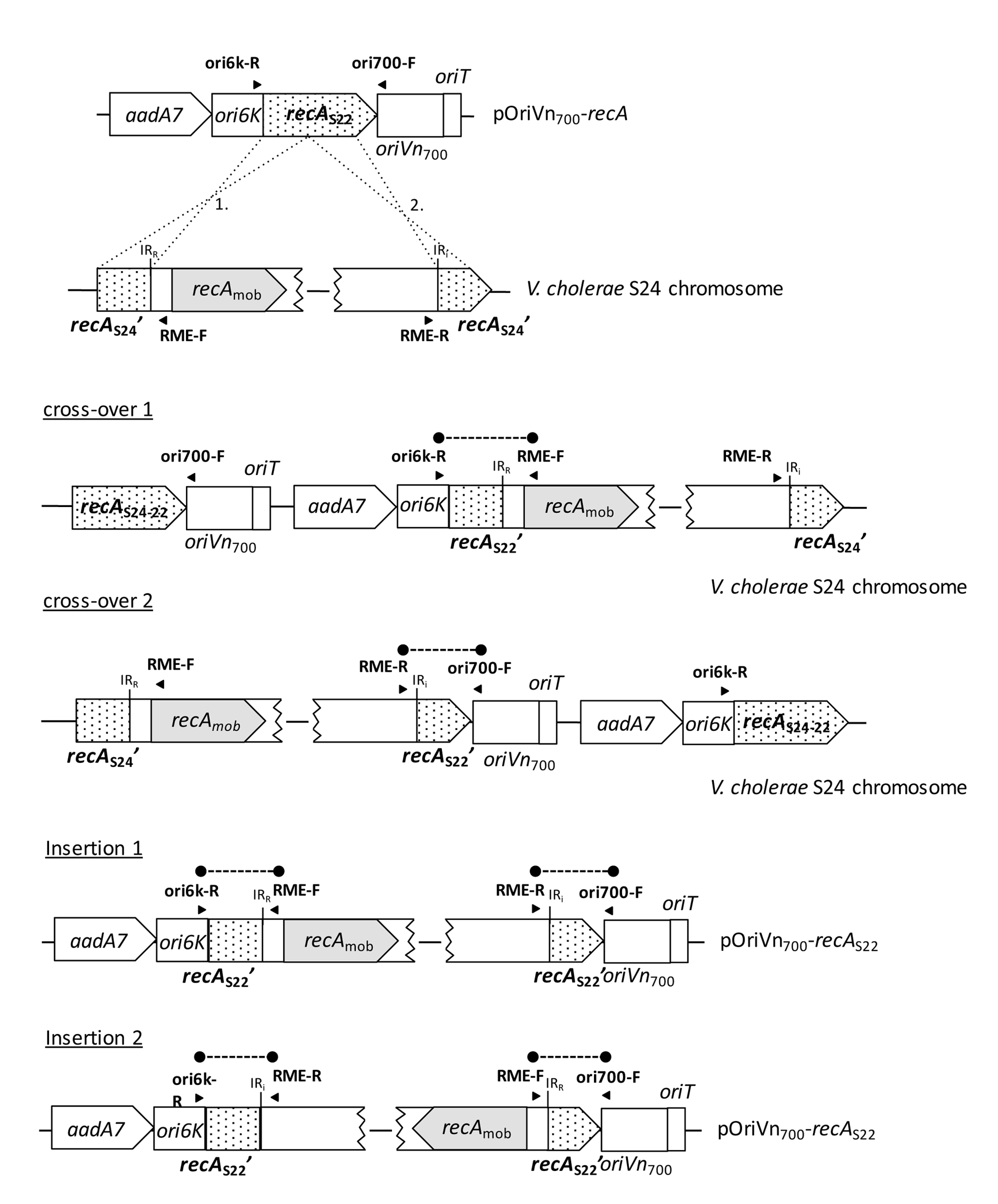

Supplement: Supplementary file 1 — Fig. S1. Possible insertion and homologous recombination events of the recA genomic island with pOriVn700-recAS22. Production of merodiploids because of homologous recombination between recAS22 and recAS24 could result in two genetic structures (crossover 1 and crossover 2). In both instances, PCR products could be generated using the vector-specific and RME-specific primers used to detect insertion of the RME into recAS22 (broken lines) in the orientation found in the V. cholerae S24 genome (insertion 1). Insertion of the RME in the inverse orientation (insertion 2) would generate products using inverse primer pairs. The inverse insertion cannot be explained by homologous recombination and indicates an integration event. [file emi0017-1090-sd1.tif]
