# Supplementary material for: A genomic island integrated into recA of Vibrio cholerae contains a divergent recA and provides multi-pathway protection from DNA damage
Source: Environ Microbiol. 2014 Jun 26;17(4):1090–102. doi: 10.1111/1462-2920.12512 (PMC4405046; doi:10.1111/1462-2920.12512)
Supplement: Supplementary file 2 — Appendix S1. Genbank file of the recA mobile element (RME); Accession Number KJ123688. [file emi0017-1090-sd2.pdf]

```
LOCUS       RecA_mobile_element_Copy32783 bp   DNA     linear       20-
DEC-2013
DEFINITION  Vibrio cholerae strain S24.
ACCESSION
VERSION
KEYWORDS    .
SOURCE      Vibrio cholerae
  ORGANISM  Vibrio cholerae
            Unclassified.
REFERENCE   1  (bases 1 to 32783)
  AUTHORS   Rapa,R.A.
  TITLE     An integrated mobile genetic element in Vibrio cholerae
provides
            multi-pathway protection from DNA damage
  JOURNAL   Unpublished
REFERENCE   2  (bases 1 to 32783)
  AUTHORS   Rapa,R.A.
  TITLE     Direct Submission
  JOURNAL   Submitted (20-DEC-2013) The ithree institute, University of
            Technology, Sydney, Sydney, NSW 2007, Australia
COMMENT     ##Assembly-Data-START##
            Assembly Method       :: NA v. NA
            Sequencing Technology :: Sanger dideoxy sequencing; Illumina
            ##Assembly-Data-END##
FEATURES             Location/Qualifiers
     source          1..32783
                     /organism="Vibrio cholerae"
                     /mol_type="genomic DNA"
                     /strain="S24"
     mobile_element  1..32783
                     /mobile_element_type="other:Genomic Island: RecA
mobile
                     element"
     repeat_region   1..9
                     /note="Inverted repeart for RecA mobile element"
                     /rpt_type=inverted
     protein_bind    98..113
                     /bound_moiety="Site for binding of LexA"
                     /function="LexA box"
     gene            204..1271
                     /gene="RME001"
                     /allele="recA"
     CDS             204..1271
                     /gene="RME001"
                     /allele="recA"
                     /note="recombinase A protein"
                     /codon_start=1
                     /transl_table=11
                     /product="RecA"

/translation="MDENKQKALAAALGQIEKQFGKGSIMKLGDNRAMDVETISTGSL

SLDIALGAGGLPMGRIVEIFGPESSGKTTLTLEVIASAQKQGKTCAFIDAEHALDPIY
```

AQKLGVNIDELLVSQPDTGEQALEICDALARSGAVDVIVVDSVAALTPKAEIEGEMGD

SHMGLQARLLSQSMRKMTGNLKASNCMCIFINQIRMKIGVMFGNPETTTGGNALKFYA

SVRLDIRRTGAIKEGDEVVGNETRIKVVKNKIAAPFKEANTQILYGKGFNRYGELIDL

GVKHKLVEKSGAWYSYQGDKIGQGKANACKFIEEHSHISQELEAKLREVLLSPVKAEG
SAVTDHEVFIDSDEATGHEEF"

```
     gene            complement(1373..2593)
                     /gene="RME002"
                     /allele="ParB-like nuclease"
     CDS             complement(1373..2593)
                     /gene="RME002"
                     /allele="ParB-like nuclease"
                     /codon_start=1
                     /transl_table=11
                     /product="ParB-like nuclease"
```

/translation="MSSKNENEHENNEIEDSIVQGLMDKYQKSPSEYLASSKQLIESS

SLRLDRKVMDEIFKSNSQYYPQLKIINLETPIRHEVVHISPTVARDMLMFSSRGKINT

DLSNRRVNRSTVKKFTEDMNEGRWCLTGEPIIIGHDGEILDGHTRLEAASKSNYGFIA

IIIWGISDALAFAHTDTGNIRSRANVLEMAGVSVDARVLSQVAMLSKSFEMTANPYAF

RGTQGTSFQPAEILDYVQGKDELAWSVNLVASLAREHKQQIQAPQATLAFAHYLINEK

LKTYEGDEIPINPDMYISNIISGLGQTSIESIEYQVRDYLESIRKESTSYALLCRLSC

IFKGWNMYFNIPVVGKKVAIRRVANYVKNADGERVPAKAAGNIREAFTIPLSPPGKTP
IRLKKQPAMKKVIR"

```
     gene            2783..3916
                     /gene="RME003"
                     /allele="Hypothetical"
     CDS             2783..3916
                     /gene="RME003"
                     /allele="Hypothetical"
                     /codon_start=1
                     /transl_table=11
                     /product="Hypothetical protein"
```

/translation="MHKFGKVKQLTSYVKVIDLFNLFSLFDIPSELEEAFQSIHSRKN

QKRIELAKTSIEAGLDNLVEVPPPCITFVVAKVLNHKSLSRSIVELEYDPLDTMIVDG

VITLFAMMQISGFSHPFEKKRISKDLTQKNCRVRQELASYPVQVNLLFSPTEPLSQKA

CITLYKKYSQTESNIYAPLIESLNAELPLNTYVKEIADDIALDSFGGMKTSSVRLSVK

DPYVTTEATMIRLVLGAIGGADYQDKNKVDLFGSGPFSAEHIDKIKPYICIFMEAWLD

SVKVQLLSNKNGFHYSTTLWQSLGLVIHNLYLEKKNLEEFAKAGAVLGRLDYSKSAKH

```
                     WGRCGALELDVLGQSYKNVTGGGRALRIALTNYLLGYLSEQEK"
     gene            complement(4204..6225)
                     /gene="RME004"
                     /allele="DNA mismatch repair;MutL"
     CDS             complement(4204..6225)
                     /gene="RME004"
                     /allele="DNA mismatch repair;MutL"
                     /note="Proetin with DNA mismatch repair MutL domain"
                     /codon_start=1
                     /transl_table=11
                     /product="MutL-like protein"

/translation="MAILQERTTGITDGSIKKRFGSIPAWKMLSEYIWNGLDAGASNV

NVTININELGGVESIEIHDNGEGIDFHNLDKNFDNWDDSSKKQVTLKGSQGRGRYSFH

KYAATATWYTRRNNENARIVIDSSCIKTYKFQPLDEIEQIKDILNNGSGTSVFLSGIT

SEKSQKIPPIESIIDSLSNEFGWKLIVCSEINISVNGTLLTPPQHKLFQDTIEIDENQ

FSSNIIQWLNKPSGEKSYNYFRDLKDELQHRTLTGFNYKNDFFISGYIQSEWFNDFQA

TSSLNNDFFVDNEKTDGSKVLDKLLKELRKKTDEIYQKFLRERAAQLVDEFESKGYFP

VYKWEQDADRLVRIEHTKKLVTSICVADPSAFNGLKAKQTKIIIALLDRLSTSSENDS

LLEILENILDLDKEHLDEFASQISRSKLDHIISTIGHLQKCDLVIQKMKYLFKEHAKD

VLETPDLQGIIEANTWLFGSQYTTIGAEEDDFSKTAKKLRDSDLEILDGDKISSSDLI

EGATIEGAKGQVDLFLARKMITVDHSTHDEFIKCTIIEIKRPSVALNKKHLAQAERYA

EVLDKHPAFNDERMRFDIVLVGTKISQNDTQINRRLKDLAGKGGAGLVSGADSRIRVY
                     VKSWSTIFNDFEVTHRSLLQKLKIQRDELEYKSKTTLVDELQVPLTEEL"
     gene            6547..9219
                     /gene="RME005"
                     /allele="Type II restriction enzyme"
     CDS             6547..9219
                     /gene="RME005"
                     /allele="Type II restriction enzyme"
                     /note="methylase subunit"
                     /codon_start=1
                     /transl_table=11
                     /product="Type II restriction enzyme"

/translation="MAVNQAKIFEQLEKLVEAPDPSEFIYGFLTAFNFPKATLTQIRQ

GGNRNVAKIKGHVGLKNKLYYFPAEGDQDIDTALDDVLADQMIAQNKIRYVLTTDFER

FLAWDTATNERLDIDFEVLHRNYSFFLPLVGLEKAILNSEKPADVKAAVKMGKLFDLI

RVHNHLNTPEDIHALNVFLTRLLFCLFAEDTGIFPKKSQFTSAIKSMTSEDGSDLDQF

LSDLFVVLNSPEGSDPRNRLPQHLIDFPYVNGGLFESDESIPKLGKKPRRILIDCGLE
```

DWSAINPDIFGSMFQAVIDVDQRARLGQHYTSYSNIMKVIQPLFLDPLRLELEKQRKS

ANGLKRLLVRLGKIKVFDPACGSGNFLIIAYKELRLLEIEVIQALMKIDQGFFISNIH

LDQFYGIEIDDFACEIARLSLWLAEHQVNKQWEEHIGPPQPALPLKTTGRVVSANSLD

IDWHTVCPNSGNEEIYVIGNPPFKGHRGRSDNQRSEMVRVFKGFKSIGLLDYVACWFW

KGAEYIKGTNAELALVSTNSISQGEQVGTLWPSIFNLGVSIHLAYPTFTWANNAKDKA

AVHVVIIGLTAKSKKGLLFERVEEDWHSRVVDSISPYLLEGGKACVLAAKAPLNSSVP

PLLFGNMPNDGGYLLLNRSERDELIAKEPEAKKWIKKVLGADEFLNSKERWCLWLKDA

TKSDLASMPLVLQRIEKVAEKRRKSSDKGTQKLADRPHQFRDLNNPSKYILMPSVSSA

RRRYVPVGVFDESVISTNLNYIIPNGTMYEFAILSSLIHNDWMRLVAGRMKSDYRYSA

SVVYNTFPWITPSDAQRKELEVLAEDILFAREDYPGKTLAELYNPDSMPLGLLSAHQK
LDTAVDRLYRKKPFNDTSERLACLLSRYEDMVTK"
gene 9247..11268
/gene="RME006"
/allele="Hypothetical"
CDS 9247..11268
/gene="RME006"
/allele="Hypothetical"
/note="Protein with helicase and phosphorothioation
system
restriction enzyme domains"
/codon_start=1
/transl_table=11
/product="Hypothetical Protein"

/translation="MKNNILQVSYNQTGQSASHNDMGMREMQARAFAERASQYLLIKA

PPASGKSRALMFLGLDKLIHQSVRKLIVAVPEVSIGGSFKDTDLMKFGFFANWTVKPE

NNLCISGSESGKTNAFKRFMESDDQILVCTHSTLRSVFDKLLPTDFDNCLVAIDEFHH

VSADENSRLGSLIDVLMKQSSAHIIAMTGSYFRGDTVPILLPEDEAKFTKVTYTYYEQ

LNGYKHLKTLGIGYHFYQGRYVNALPSVLDPTRKTIIHIPNVNSVESTKDKYTEVDSI

LEVLGDVMMQDPETGIYQVKCAKTGKTLLVANLVDDSDMRPKVQAYLRNIESAEQMDI

IIALGMAKEGFDWPYCEHVLTIGYRSSMTEIVQIIGRATRDSEGKSHAQFTNLIAQPD

AQDDDVKVSVNNMLKAITTSLLMEQILAPSIQFKPRSQWNGEDLPANTLIVDDTTTSV

SQKVLDILNRGKDEILSALLSKESVVKGAIADTMPAEVINEIELPSVIQTLYPDLEEH

EIEQIRTGVLHSLYIGQKGGLIDGKDLPEDAVIEDGGASYGNGHKSKDNGESGTSNQF

```
IKMGDKFINIESLDIDLIDSVNPFHGAYEILSKSVDAAVLKTIQQTVRASQANVSEEE
                     AVMLWPRIKAFKVEHQREPSLNSSDPIEQRYADVLAYIRRMKQQRMAQQ"
     gene            11284..12531
                     /gene="RME007"
                     /allele="Hypothetical"
     CDS             11284..12531
                     /gene="RME007"
                     /allele="Hypothetical"
                     /note="YeeC-like nuclease domain-containing protein"
                     /codon_start=1
                     /transl_table=11
                     /product="Hypothetical Protein"

/translation="MIRLRSSKATEKFDLDDIFSEQDDLGLLDVAPLRAKIPTGNILA

NQFEEISNFYEQNGRVPRSDAQSFYEKRLARRLNAFKSNPEQCDVLSEYDRYSLLDSD

LAAQENVQRDKVEDKSLPTELDKSELVTSLDDIFDDDDDGLLEFDAPHLFTKTHVPAE

KKSQPNEIARRQPCAEFHRFSPIFETIQQEIRSGSASLERFRHELQMRVGDVFILNGL

IGYVHSAGERLEGYSSYNARLHLIFENGTEMHMLFQSLTHGLVRDERGCKIIREGESL

EPDDTPVPAGLVYVLATKSTDSALAPYKANLYKVGFTDGKVEERIKYAEKDKTFLEAP

VRVVMTTECYNIDAHKLETLIHGFLGHRRLNVTLKGHAGQYSPREWFYVPLNTVLEVI
                     GYILDGTISQYRMDNTTGKVVKK"
     repeat_region   12870..12876
                     /note="Direct repeat for insertion of ISVuv4"
                     /rpt_type=direct
                     /rpt_unit_seq="ccttaag"
     mobile_element  12877..14083
                     /mobile_element_type="insertion sequence:ISVvu4"
     gene            13036..14025
                     /gene="RME008"
                     /allele="Transposase for ISVvu4"
     CDS             13036..14025
                     /gene="RME008"
                     /allele="Transposase for ISVvu4"
                     /codon_start=1
                     /transl_table=11
                     /product="Transposase"

/translation="MQKNSVMYLEPVRLWGYQEILSTVXQELVETGGIDALINRSRRA

PNLKNRVDSETEQAVIKYAIDFPAHGQVRTSNELRKLGVFISPSGVRSIWLRNDLENF

KKRLIALEKQVAENGIILTDEQVAALERKKHDDEACGEIETAHPGYLGSQDTFYVGNL

KGVGRIYQQTFVDTYSKVAFAKLYTTKTPITAADILNDKVLPFFEAHELPMLRILTDR

GTEYCGRVEQHDYQLYLAINDIDHTKTKAMSPQTNGICERFHKTILNEFYQVTFRKKL
```

```
YGSIEELQKDLDEWMDYYNNHRTHQGKMCCGRTPIETLEDGKSIWAEKNLAQI"
     repeat_region   14084..14090
                     /note="Direct repeat for insertion of ISVuv4"
                     /rpt_type=direct
                     /rpt_unit_seq="ccttaag"
     gene            complement(14114..14731)
                     /gene="RME009"
                     /allele="Hypothetical"
     CDS             complement(14114..14731)
                     /gene="RME009"
                     /allele="Hypothetical"
                     /codon_start=1
                     /transl_table=11
                     /product="Hypothetical Protein"

/translation="MPMFKYHLDTTKKLSVNGQGFSVLQVYTDTKVTNSQLFINVNDL

VENSPLTRGEVNEHVANASEEQVIIDQEQTLIRVSSALKLNDPKLRDVDPNVRSQAQQ

FEQVIDKINMMPKLNEERAIASETVKTKSTKAKQDYKNQRVIQGLGNVCEKTNQPIPQ
                     GDNLHIHHDPREADFPELAAEEASLSAIGSTVHSEGHKNDNNPFN"
     gene            complement(14734..15192)
                     /gene="RME010"
                     /allele="Hypothetical"
     CDS             complement(14734..15192)
                     /gene="RME010"
                     /allele="Hypothetical"
                     /codon_start=1
                     /transl_table=11
                     /product="Hypothetical Protein"

/translation="MFMSDTNLPIELKPLSELIDVKPIEISPDLDEKLTENNQVLVSK

SIMKIDHQTKTPTPFFSVDSLVSCIGTDRKPFRELMADAADGEVIKINNKYLIRSDLT
                     KQFLQERSEQPRSCGERARIEATRSIVNEVGKLDYEQVIALLNNKVQGDE"
     gene            complement(15199..15609)
                     /gene="RME011"
                     /allele="Hypothetical"
     CDS             complement(15199..15609)
                     /gene="RME011"
                     /allele="Hypothetical"
                     /note="DnaJ domain-containing protein"
                     /codon_start=1
                     /transl_table=11
                     /product="Hypothetical Protein"

/translation="MTSLYVVVFALLLTVVQLLFLLRKYKKKIQELQSTYVESSTTAE

EADLQVNLVRTSTDDMAYFKSENDRILFLLLEVDGKRRNQLLGITSEMYEDEDAAKKW
                     YKSLSNKVHPDKNDDPRAAEAFDKLKQLYNKVTY"
     repeat_region   15890..15896
                     /note="Direct repeat for insertion of ISVuv4"
                     /rpt_type=direct
```

```
                     /rpt_unit_seq="cctaaat"
     mobile_element  15897..17103
                     /mobile_element_type="insertion sequence:ISVvu4"
     gene            16056..17045
                     /gene="RME012"
                     /allele="Transposase for ISVvu4"
     CDS             16056..17045
                     /gene="RME012"
                     /allele="Transposase for ISVvu4"
                     /codon_start=1
                     /transl_table=11
                     /product="Transposase"

/translation="MQKNSVMYLEPVRLWGYQEILSTVIQELVETGGIDALINRSRRA

PNLKNRVDSETEQAVIKYAIDFPAHGQVRTSNELRKLGVFISPSGVRSIWLRNDLENF

KKRLIALEKQVAENGIILTDEQVAALERKKHDDEACGEIETAHPGYLGSQDTFYVGNL

KGVGRIYQQTFVDTYSKVAFAKLYTTKTPITAADILNDKVLPFFEAHELPMLRILTDR

GTEYCGRVEQHDYQLYLAINDIDHTKTKAMSPQTNGICERFHKTILNEFYQVTFRKKL

YGSIEELQKDLDEWMDYYNNHRTHQGKMCCGRTPIETLEDGKSIWAEKNLAQI"
     repeat_region   17104..17110
                     /note="Direct repeat for insertion of ISVuv4"
                     /rpt_type=direct
                     /rpt_unit_seq="cctaaat"
     gene            complement(17211..17645)
                     /gene="RME013"
                     /allele="Transcriptional activator SoxR"
     CDS             complement(17211..17645)
                     /gene="RME013"
                     /allele="Transcriptional activator SoxR"
                     /note="Redox-sensitive transcriptional activator
SoxR"
                     /codon_start=1
                     /transl_table=11
                     /product="Transcriptional activator SoxR"

/translation="MIRNDYLTVGQLSERSGVATSALRFYETKQLITSFRTNSNQRRY

LPSMLRRVALIQVAQSVGFTLEEIRYELSTLPMNKTATKQDWDRVAKKWQQDLDKKMV
                     KIQSLKDNLSGCIGCGCLSMKKCHLFNPEDGLYEQGAQRTIR"
     gene            17963..18844
                     /gene="RME014"
                     /allele="Drug/metabolite transporter (DMT)
superfamily"
     CDS             17963..18844
                     /gene="RME014"
                     /allele="Drug/metabolite transporter (DMT)
superfamily"
                     /note="Permease of the drug/metabolite transporter
(DMT)
```

```
                     superfamily"
                     /codon_start=1
                     /transl_table=11
                     /product="Permease of the drug/metabolite
transporter
                     superfamily"

/translation="MLKNYFTLIAIGLIWGSQFIFQETALEAFTPVWIGTFRAVLGAT

TLFIICRIMRICSSSKQWKLYTVIGFLEATVPFVLVPFAQKELSSSVTAILMGTLPFY

ALLLAPLFIRNTKITKGNLFSITIGFFGLVVLFYPDLISSSQRIELVSVLAVLLAAVC

FAIALLLLNRVQREHPLIVARNVLTMASIQLIIVALITTTRMTYKAPSPSIFIAVIYL

GVMCAGVVYYLYMTSVKNAGAVFTSMTNYLVPAVGVLIAALVTNESIQTTTWLALGII
                     LSALLLNQMVEKYEQKT"
     gene            complement(19225..21105)
                     /gene="RME015"
                     /allele="Hypothetical"
     CDS             complement(19225..21105)
                     /gene="RME015"
                     /allele="Hypothetical"
                     /note="chromosome segregation ATPase-containing
protein"
                     /codon_start=1
                     /transl_table=11
                     /product="Hypothetical protein"

/translation="MTQYKPFLLVKRLVITKGKSIAYDEKFTAGVNIIRGVNSSGKST

ISDFIFYGLGGDLTKLKNEAKQCSFVFVEASLSGKVFTLKREIAEGGRKGMDIFSGDY

ESAYVASVTDWIRYPYNANTKESFYQALFKELGMPYSKSDDKNSITMHQLLRMLYVDQ

MTSPDRLFKFDKFDSPNKRQAIGELLIGLSDFELYEKRVRLQSLKLALENRIKEIKTI

HSFLGGTIKSVSEINGEIEEKRKEIDALELEVESFSSPSEDGCTEDEVLKNLIVEVQE

ARGKYTASQQEIAKTSFEINDSQMFIESLSRRVKALKETQDTINALSDVAFNHCPACQ

TEVQARPTGCSLCGATKPESENNIDPTFKVRKEIEFQIAESILLIEKKQIRLDEQKVE

SNQLETKLGELERDLEIIRKPQRAVNIQLRQKLSEIGGLRNEIRTLNNSKKEFAKLYG

LYDERDTLQTDFNTLNDEITRLTQRMENELKAKKRKLSEATLSILKADAGHEEIFVDG

SKVEFDFAEDRVTIDDRALFSASSMVYLKNAFRLAMLKCSCEDSSYLYPRFLLMDNIE

DKGMEEERSQLFQREIVKLSNSLDVEHQIIFTTSMIDSDLNHSEYCVGDFYNMHNKTL
                     KV"
     gene            complement(21598..22080)
                     /gene="RME016"
                     /allele="Hypothetical"
```

```
     CDS             complement(21598..22080)
                     /gene="RME016"
                     /allele="Hypothetical"
                     /codon_start=1
                     /transl_table=11
                     /product="Hypothetical protein"

/translation="MYRQDHRLRELVEEHEKEIQKDEQYKEFSSQLNNFLERKVEGKL

RDLGQKLTDGNRDFLLDYAMDVKERVSKKIMQFSHYKSAQELYTYILTNIRTTFLHEI

SPRIKSGDFKVYQINDLVEEKIIQPVLESVHGCSLNIDKDELYGLLYILTGNCYVEWD
                     "
     gene            complement(22683..23939)
                     /gene="RME017"
                     /allele="Error prone, lesion bypass DNA polymerase V
                     (UmuC)"
     CDS             complement(22683..23939)
                     /gene="RME017"
                     /allele="Error prone, lesion bypass DNA polymerase V
                     (UmuC)"
                     /note="Error prone, lesion bypass DNA polymerase V
(UmuC)"
                     /codon_start=1
                     /transl_table=11
                     /product="UmuC"

/translation="MFALVDANSFYCSAEQVFRPDWRGKPMIVLSNNDGCIVAANRQA

KEVGIEKFKPYFQVKALCEQKGVIALSSNYELYADLSAKMMQVIGRFAPEQHIYSIDE

SFLSFEHSFPAIRSLKEHGMKLRRAVWRECRLPVCVGFGQTLTLAKVANHAAKKIEGY

NGVCVLDSERERKAVLGQLHVSEVWGIGRKLTHRLEMMGINTALKLANYPPALIRKEF

NVEVERTVRELNGQKCKGWDAARADKKQIFSTRSAGQRITDLESLQQALCKHANIASF

KARKQKSLCRVMLCFANSSPFDSPPVARRAVHRFAYPTADVTLITQAASRLAEQLFQQ

DVRFYKIGVGLIDLVDGQHEQPDLFNLTPNNPKLMNVYDTLNNRFGNDAIFLAAQGIT
                     QKWAMRREMLSPQYTTRWQDLPKIKC"
     gene            complement(23939..24199)
                     /gene="RME018"
                     /allele="Error-prone repair protein (UmuD)"
     CDS             complement(23939..24199)
                     /gene="RME018"
                     /allele="Error-prone repair protein (UmuD)"
                     /note="Error-prone repair protein UmuD"
                     /codon_start=1
                     /transl_table=11
                     /product="UmuD"

/translation="MQDVGIFDKDLLIVDRSLDVQDFDIIVANLNGEFICKQIDLNRR
                     LLLSANERYQPVPIHEFDQFSLEGIVTRSIRCHRVSPLLRTR"
```

```
     protein_bind    24359..24374
                     /bound_moiety="Site for binding of LexA"
                     /function="LexA box"
     gene            24984..25739
                     /gene="RME019"
                     /allele="Hypothetical"
     CDS             24984..25739
                     /gene="RME019"
                     /allele="Hypothetical"
                     /note="Multi domain XerS site-specific tyrosine
                     recombinase XerS"
                     /codon_start=1
                     /transl_table=11
                     /product="Hypothetical protein"

/translation="MLWSKMPSKWIAEGILRESFSGGRSVSDDIAALKLFIVLCFLAK

QVNRSSGSSVDDVLEIRATYDQLTDMCSLSRSLVSRGLKKLHITGLLSSAGVRTKTYT

FIQGVYRGWCKLPKRALVKNEEEIPAMKAFLNRYSHERDALKCFLYILASRSNSRTYV

DLSRGTIAKKTGVSLDAIDGAIGFLQSTSLISKVEDKGFLANSIRRDLSERLHRYWVI
                     GSSSLNYKTYSVESEDSIFVREPSLYFMENNRR"
     gene            complement(26001..27461)
                     /gene="RME020"
                     /allele="Hypothetical"
     CDS             complement(26001..27461)
                     /gene="RME020"
                     /allele="Hypothetical"
                     /codon_start=1
                     /transl_table=11
                     /product="Hypothetical protein"

/translation="MKLDSLKIDVKDCLPSDESDPNISWESSLFLHGNGEVNWFATDF

LMSDAIPNRSKKTAKGMIRYFLEYLECYENWKYNDIQGRPFPIGLITDSHLYDYVQYI

EDDIGLNRNAIANRVRMALRFLEYVQKYYHLSYTLIAIANTDGEYFTKGLVNAERKIS

PYGKRYLHHDCIPHCESYGSRSPITDTAIESLYDDLDILEAEGDLYRFEFFSTLISLL

EATGIRVSEAANIDTHTIEVLRAQVNASLSGKAIGLDEIISLNKLTINTQSLQAAQAI

YRKSALGSANDQLIWIKIKTTKGKNKDKFRIIPISFTTAQYLIRFYDDYIVNELDRIS

KGLAKVNRAKFGKLFVHPSSHLPMSGIMISRLFYDVFSRKFKSKHKRSPHLFRHRFIT

LLVLQQLKALKTNIGGTQLAILILNRIKGLTGHASIKAMLHYVELAEAELYEDEDESE
                     VFDRVTRDHLVAELGAEHVAEIEAGLRLKKAKQAFI"
     gene            complement(27465..28181)
                     /gene="RME021"
                     /allele="Hypothetical"
     CDS             complement(27465..28181)
                     /gene="RME021"
```

```
                     /allele="Hypothetical"
                     /codon_start=1
                     /transl_table=11
                     /product="Hypothetical protein"

/translation="MKMNLKRELSPNHNGPKGRRDTLEQVKLRVILLNDFIENGVPDG

FTAHISLKALLAYSDSGKIQSRSYPAIYNKKKILVKEIDPSFTGSASSVVDCKDYLLL

KIEELRSKIETTSTPSEQVTEAENECNNDDDPKVKTKGELRSALKEQAELIESLAREI

LRQRSANNTLISLIKERDKYASRTLKSYYEDHQTELCKVRTVIKPALRETISNLNKLS
                     HEFDDIFAESEENIVSIFGK"
     gene            complement(28165..30198)
                     /gene="RME022"
                     /allele="Phage integrase"
     CDS             complement(28165..30198)
                     /gene="RME022"
                     /allele="Phage integrase"
                     /note="Protein containing phage integrase family
domain
                     protein"
                     /codon_start=1
                     /transl_table=11
                     /product="Phage integrase"

/translation="MTLININNVKLREGADWSYKSDDTSKWHFAWLHGNFSDSVWHID

NGSGKSGSDLPYISFNDYIPTTGDTLDEDKYLIVTNTIRKVIALNRTGHLSTETKNSS

LLTRFSSMNSNAIALKILALFLIKEYGESAVSEEGFNLLTTTDIRKFHLEIATGRADK

ATGLTDIILEKISTMDRESAIQLFDINVESKNNELPVGEFLQRIGMQNTSISDFTMNL

ISDRLVERFPDLSLTITGNNNGKKSEFPNSCPKLFTGKENNISSSTFDALTKGPLLLK

RYSNYLPELENYCNPIAKVSKSFKKNHIRSKGRTPNIPTKTALHYLNEAIRIVVVYGE

SIVATKINCELQLNKIHKNNPSYLRSHIFEGRYPVNVAIPKNKFTQDFKVTRYNELQK

GSTPKKLRENVTVLFGYRILLAATYILTHTFCIKRTTEILELKESNLELGFWGGYELF

FGIRKAAPTENSILVTGRPIPHVLFEGISYLAEANEHYYCENEDPFLFPEKYTSAAEG

LPPKNSKMSRFAMSNILKDFGDFIQVPTEMVNGIESRFYLSRTHVLRRFAARAFYALT

DISDFPAISWLMGHRSTQETWRYLLEDVSNEELSEEEAQGVLDAIYKKGVDTSQVENV

IKTELNIKFNNQSDELAKEFIKEQLLSGSKIYSYTDESGKVIVWMEVGNEDES"
     gene            complement(30185..32131)
                     /gene="RME023"
                     /allele="Hypothetical"
     CDS             complement(30185..32131)
                     /gene="RME023"
```

```
                     /allele="Hypothetical"
                     /codon_start=1
                     /transl_table=11
                     /product="Hypothetical protein"

/translation="MNIKEHTINGMHCQKIMAHQYAVNMSISITKILSGKSDSYKRRT

TRMLNEFFTFCYKNNLHMKTGADVSLCYSAWQEFYFSGAVKKQPHTLLSDSRSLWSAL

LRLCKRSVNGLPKTNHITPPRRLNTLVFSDTTNSQAKVLGGFQLSTNTTGESLTLILE

TDVDAFLGNLISTMKKHRDVIYKVSKSYLIDACDRFTFGQEAMEKIDASVFENNPTLL

HPSLLNGKGSRLSLFNPDLPNGECGKTNLLAYIGHCKNGLVDRHFLGGNNHLYRFTSN

QYELREHFGLSTLSAVACCNIIITESGINVDSLRKLALSAQGSINKIFEPSQHGFRVS

YHKARAKEHLKRNLRHATDTPFIEKAFNYIIEATSYHRSLASGNDANRLFLYQTATAT

HKVMPMSDMAFKSGFVRLLVEAKKILKNTPNWCEGVTPECIDEVLMAAPTAKKLRATE

GVIRWYESGGNPAVAAKYLGNSEAVSIRNYLPQELQLAVYNQRVRRFQHVLISSATDG

QKYQIKALKLKDETELTEYLSRLDERIPHWRSVIETISDNNRNSSTASDIKITLDICP

ENIALMKACHEIAMQRIDSANHTEDSISELSFVYQGLTGYLNTHPDRRLMRTLQKGNL
                     LYEKKTGSLLDLKIIKQEVQYDSN"
     repeat_region   32775..32783
                     /note="Inverted repeart for RecA mobile element"
                     /rpt_type=inverted
                     /rpt_unit_seq="cgataaaat"
BASE COUNT     9474 a   6453 c   6605 g  10250 t      1 others
ORIGIN
        1 attttatcga agagagatta gctgcttagt ataaggtaga actagtttga
ttagtgcctt
       61 ttaaaacaat ccgctcataa gtcagtaatg cttcactcag ttataaataa
ttgaatgtat
      121 attcatttta gatttgaatt agaaagacta aattgaatat gcattacgtg
cataactaaa
      181 tagaattcag agggtatata aagatggatg agaacaagca gaaagcgtta
gcagcagcgc
      241 tcggccaaat tgaaaaacaa ttcggtaaag gctctatcat gaaattgggt
gacaaccgag
      301 caatggatgt tgaaacaatt tctacaggtt cactttcttt agatattgct
ctcggtgcgg
      361 gtggtttacc tatgggtcgc atagtagaga tctttggccc tgagtcatca
ggtaaaacaa
      421 cgcttactct tgaggtaatt gcttcggcac agaagcaagg taagacttgt
gctttcattg
      481 atgcagagca tgcgctagac ccgatttatg ctcaaaaact aggtgttaat
attgatgaat
      541 tacttgtttc tcaacctgat acaggtgagc aagctcttga gatctgtgat
gcgcttgctc
```

601 gttcaggagc tgttgatgta atcgttgttg actctgtagc agcattgaca ccaaaagctg
661 aaattgaagg tgaaatgggc gattcacata tgggcctcca ggccaggctt cttagccagt
721 cgatgcgtaa aatgactggt aatttgaagg cttcgaattg catgtgtatc tttattaacc
781 agattcgtat gaagataggt gttatgtttg gcaacccaga aacaactact ggtggtaatg
841 ctctaaaatt ctacgcttct gttcgtctgg atattcgtcg tactggtgcg atcaaagaag
901 gtgatgaggt cgtgggtaac gaaacgcgta tcaaagttgt caagaacaag attgcagcac
961 catttaaaga ggcaaacacg caaatccttt atggcaaggg cttcaaccgc tatggtgagc
1021 taattgacct tggcgttaaa cacaaactgg tcgaaaagtc tggcgcttgg tacagctatc
1081 aaggtgataa gatcggtcag ggtaaagcaa atgcctgcaa gtttattgaa gagcatagtc
1141 atatttctca agagttagaa gctaaactgc gtgaggtact attatctcct gtcaaggctg
1201 aaggttcagc ggtcaccgat catgaagtgt tcatcgactc tgatgaggca actggacacg
1261 aagagtttta atctttagtt tgacacctaa aggtaaattc tacaaaaagc gccctgcata
1321 atgggcgctt ttacatttca tatattcctt aacttattgt ttagggctgt ttttatcgaa
1381 tcaccttctt catagcaggt tgctttttta acctaatagg cgtctttccg gggggggaaa
1441 gaggtattgt gaaagcttct cttatatttc ctgctgcttt tgctggaact cgttcaccat
1501 cagcattttt cacatagttc gctacacggc gaattgctac ctttttgcct acaaccggaa
1561 tattaaaata catgttccat cctttgaaaa tgcatgataa acggcatagg agtgcataag
1621 atgttgattc tttccttatc gattcaagat agtctcgaac ctgatattca atactctcta
1681 ttgacgtttg ccctaaccct gagatgatat tagatatgta catatcagga ttaattggaa
1741 tttcatcgcc ttcgtaggtt tttagttttt catttatgag ataatgtgca aatgccaaag
1801 ttgcttgagg tgcttgaatc tgttgcttat gttctctagc taaagaagca accaaattaa
1861 ctgaccacgc taactcatct ttaccttgta cgtaatcgag tatttcagca ggttggaatg
1921 aagtgccttg agtacctcta aatgcatacg gattggctgt catctcaaaa gattttgaaa
1981 gcattgcaac ctgagacagt actctagcat ctacgctaac acctgccatt tcaaggacgt
2041 tagctctgga gcgaatatta cctgtatctg tgtgagcaaa tgctagagca tcagagatcc
2101 cccatataat gatcgcaata aatccataat ttgattttga tgcggcctct agtcttgtat
2161 gaccatccag tatttcacca tcatgaccga taatgatcgg ttctccagta aggcaccacc

2221 gtccttcatt catgtcttcc gtgaactttt tgactgtact tcgattaaca cgacgattgc
2281 ttaaatcggt attaattttt cctctacttg aaaacatgag catgtctctg gcgacggttg
2341 gtgaaatatg cacaacctca tgtcgaatag gagtctctag atttatgatc ttgagttgag
2401 ggtaatactg ggagtttgat ttaaagattt catccattac cttcctatct agtctgagac
2461 tggatgattc aattaactgc tttgagctgg ctaggtattc actgggagat ttttgatatt
2521 tatccatcaa cccctgaaca attgaatcct ctatctcatt gttttcatgt tcattttcat
2581 ttttgctact catcatttac ctcaaatctt tcaggttcat agatcaaaaa ccttccaatt
2641 ccacaaagtt agtgtttaat tgttgatcgg acggggcgtc cgatcaatat ttattttaat
2701 ctcaatttag aatgcaaact caacatagtg aggggttcat tcattgaaaa agacatcagt
2761 gaggctggaa ggcttatgct caatgcacaa gtttggtaaa gttaaacaac taacttcata
2821 cgttaaagtc attgatttat ttaacttatt ctcgctattt gacataccaa gtgaacttga
2881 agaagcattt caatcgatac acagccgaaa gaatcaaaaa aggatcgagc tagcaaaaac
2941 ttctattgaa gcgggcttag ataacttagt tgaggtgcct ccgccatgta tcacatttgt
3001 tgtggcgaaa gtcttaaatc ataagagttt aagtagaagt atcgtagaac tagaatacga
3061 tccactcgat actatgattg tggatggtgt gattacgttg tttgccatga tgcaaattag
3121 tggttttagc catccatttg agaaaaagcg tatatctaag gatttaactc agaaaaactg
3181 tcgagttagg caagaactcg caagttatcc cgtacaagtt aacttgttgt tctcacctac
3241 agaaccattg tcacaaaagg catgtatcac tctttataaa aaatacagcc aaactgaaag
3301 caacatttat gcacctttga ttgaaagtct aaatgcagag ttgcctctga acacttatgt
3361 aaaagaaatt gcagatgata tcgctcttga ttctttcggg ggaatgaaaa cgtcgtcagt
3421 taggctgtcc gtaaaagacc catatgtgac tactgaggca acaatgattc gtctagttct
3481 tggtgcaatc ggtggtgcag attaccaaga taaaaacaaa gtcgatttgt ttggttctgg
3541 gccatttagt gccgagcata tcgataaaat aaagccttat atttgtatat ttatggaagc
3601 gtggcttgac tcagtaaagg ttcaactgct ttcaaataaa aatggctttc attactcaac
3661 gacgctttgg caatcactag ggctagtcat acataacctt taccttgaga aaaaaaactt
3721 agaagagttt gctaaggctg gggctgtttt aggtcgtctg gattactcca aatcagctaa
3781 gcactgggga aggtgcggtg cgctggaact tgatgtgttg ggacaaagct ataaaaacgt

3841 tacaggaggt ggtagagctt tgcgtattgc cttaactaac taccttcttg gatacttatc
3901 agagcaagaa aagtaagaat gtttctattg tccactcatg gtcaatgatg gtcaatggtg
3961 aacaattgct taatttaaaa gaggaaaggt atgagcttta aggcagttaa atgacaacct
4021 aacatttagt taatgtttga attgtcgagg attttattct tgaagtacaa gtaagagctg
4081 tctctaggtt tgttctgtaa agtatttgcc cacccccccc aaaaaaaaag gcaagctatg
4141 gggtgggcta tgtagttgag ttgaagccgc gcctgtgaac aaacaacctc aacactagtg
4201 aaatcacaat tcttcagtta gaggaacttg taattcatcg acaagagtgg ttttggattt
4261 atactccagc tcatctcttt gtatttttag cttctgcaac aaagatctgt gggtaacctc
4321 aaaatcattg aatatcgtag accaactttt gacgtataca cgtattcttg agtcagcccc
4381 agaaactaga cctgcaccac ctttacctgc aaggtctttt aagcgcctat tgatttgggt
4441 gtcattctga gagatttttg taccaactag aacaatatcg aatcgcattc tttcatcgtt
4501 aaaagctgga tgcttatcta aaacttcagc atagcgctca gcttgagcca aatgtttctt
4561 atttagagcg acacttggcc tctttatctc tatgattgta cattttatga actcatcatg
4621 ggtcgagtga tcaaccgtaa tcatcttacg agcaaggaat agatcaacct gccccttgc
4681 cccctctatg gttgcccctt caatcagatc gctggagcta atcttgtccc cgtctagtat
4741 ttctaaatct gaatctctca gttttttgc tgttttagaa aaatcatctt cctcagctcc
4801 tatagttgta tactgagagc caaataacca tgtgttagct tcaataatac cttgtaaatc
4861 aggagtttcg agaacatctt tagcatgctc tttgaataga tatttcattt tttgtatgac
4921 taagtcacac ttctgaaggt gtccaatagt gctaattatg tgatcaagtt tagatctact
4981 gatctgagag gcaaattcgt ctaagtgctc cttgtctaag tctaaaatat tttccaatat
5041 ttccagtaag ctatcatttt cactagacgt tgagagtcta tccagcaaag cgatgataat
5101 tttagtttgt tttgccttta ggccattgaa agcggatggg tctgcaacgc agatgctggt
5161 gactaacttt ttagtatgct caattcttac caacctgtct gcatcttgtt cccatttata
5221 tactggaaag tatccttttg actcgaactc gtctacaagt tgagcggctc tttctctcag
5281 aaacttctga taaatttcat cagtcttttt gcgcaactct tttaatagtt tatctagaac
5341 tttagaccca tcggtttttt cattatcaac gaaaaaatcg ttattgagag atgaggtggc
5401 ttgaaaatca ttaaaccatt cagactggat gtaaccgctt atgaagaaat cattttgta

5461 gttaaaacca gtaagagttc ggtgttgcag ttcatctttt aagtccctaa agtagttgta
5521 agatttttcc cctgatggtt tgtttagcca ttgaataata tttgatgaaa actgattttc
5581 atcaatctct atagtatctt gaaatagctt gtgttgaggg ggtgttagta gagtaccgtt
5641 gactgaaatg ttgatttcgg agcaaacaat tagcttccag ccaaattcat ttgacagaga
5701 atctatgata ctttcaattg gtggtatttt ttgagatttt tcactagtga tgcctgaaag
5761 gaaaactgat gttccactac cattattaag aatgtctttt atttgttcaa tttcgtctaa
5821 tggctgaaat ttgtaagttt tgatacagct tgagtcaatt acaatacgcg cattttcatt
5881 atttcttctg gtataccaag tcgctgtggc ggcatacttg tgaaacgaat aacggcctct
5941 gccctgacta cctttgagcg tcacttgttt ttttgaagaa tcgtcccaat tgtcaaagtt
6001 tttatctagg ttatgaaagt ctataccctc tccattgtca tggatttcaa ttgactcaac
6061 gccaccgagt tcattgatgt taatagttac attgacattt gaagcgcctg catccaaacc
6121 attccagatg tattcactca acattttcca agcaggaatt gaaccaaatc ttttttttat
6181 cgaaccatct gtaatgccag ttgttctttc ttgaagtatc gccactgact aaccctttct
6241 tcagtacatt ccacacaaat catcttaacc taagtcacgg gaattattaa ttataattga
6301 tgaacctttg ctgtccgact aggccatgta ccatctatta cagcacctgt taagcacata
6361 tcacggaagc atcaaagatc cttccacaaa aggcatggtt acgacagtgg aaaattttct
6421 agcagtagca atcccctttg tttctgtgtg gtagatattt gatatgaatc tcaatgatta
6481 aagtgtgttt tatttgtata atttcccgca tttatggcgc gattcgcaaa ccaatttagg
6541 aacaccatgg ccgtcaacca agcaaaaatc ttcgaacaac tagagaaact tgttgaagct
6601 cctgacccta gtgagttcat ctatggattt cttacagcat tcaatttccc taaagcaacg
6661 cttactcaaa tacgtcaagg tggcaaccgt aacgttgcca agattaaagg tcatgttggg
6721 ctaaagaata agctgtatta cttcccagct gaaggcgatc aagatatcga tactgcttta
6781 gatgatgtcc ttgcagacca aatgatcgcc cagaataaga ttcgctacgt cctgaccact
6841 gactttgaac gtttcttggc ttgggatact gccactaatg aacggctaga tattgacttt
6901 gaagtcttac accggaatta cagtttcttt ttaccactgg tagggttaga gaaggcaatc
6961 cttaacagtg aaaagccagc ggatgtaaaa gcggcagtca agatgggtaa gctgtttgac
7021 cttattcgcg ttcataatca tttgaacacc cctgaagata ttcatgcact gaacgttttt

7081 ctgactcgcc tgctgttctg cttatttgca gaagatactg gtatttttcc aaagaaaagc
7141 caatttacgt ctgcgattaa gagcatgacc tcggaagatg gtagtgattt agatcagttc
7201 ctttctgatt tgtttgtggt tcttaatagc cctgaaggaa gtgacccacg taatcgtttg
7261 ccgcagcacc tgattgattt cccatacgtt aacggtggct tatttgaaag tgatgagtcc
7321 attccgaagt tagggaaaaa accccgccgt attctgattg actgtggact ggaggattgg
7381 agcgctatta accctgatat cttcggaagt atgttccaag ctgttattga tgtggatcag
7441 agagcacgac tagggcagca ctatacctct tacagcaata tcatgaaggt gattcaaccg
7501 ttatttctag acccttacg acttgaatta gaaaagcagc gtaaaagtgc taacgggtta
7561 aaacgccttc ttgttcgatt aggtaaaatt aaagtttttg accctgcttg tggttctggc
7621 aactttctga tcattgctta taaagagtta cgtttgcttg agattgaggt tattcaggca
7681 ctgatgaaaa tcgaccaagg cttctttatc agcaatatac atctggatca gttttatggc
7741 atcgagatcg atgactttgc ttgtgagatc gcacgtcttt ctctttggtt ggctgagcat
7801 caagtcaaca aacaatggga agaacacatc ggccccccac agccagcact acctcttaaa
7861 acaacaggta gagttgttag cgctaatagt ttggatattg attggcatac agtttgccct
7921 aactctggga atgaagagat ctatgtgatt ggtaaccctc cgtttaaagg gcatagagga
7981 agaagtgata atcagcgttc tgaaatggta agagtattta aaggctttaa atctataggt
8041 ttattagatt atgtggcctg ttggttttgg aaaggtgctg agtatattaa gggtacgaat
8101 gctgaattag cgcttgtgtc gactaattca atatctcaag gagaacaagt tggtactctg
8161 tggccaagta tttttaattt gggagtttcg attcatttag cttatccaac tttcacttgg
8221 gcaaacaatg ctaaagataa agctgcggtg catgtagtaa ttataggatt aaccgcaaaa
8281 agcaagaaag ggctattatt tgaacgagtt gaagaagatt ggcattcaag agtcgttgac
8341 agtattagcc catacctatt ggaaggaggg aaagcttgtg tgttggctgc aaaggctcct
8401 ctaaatagtt cagttccacc cctgctattt gggaatatgc ctaatgatgg tggatactta
8461 ctgctaaatc gaagtgaacg agatgagtta atagcaaagg aaccagaagc taaaaaatgg
8521 attaaaaaag tactaggtgc agatgaattt ctaaattcaa aagaacgctg gtgcttatgg
8581 ctaaaagatg caacaaaatc tgatttggca tcaatgcccc ttgtactaca aaggattgaa
8641 aaagtagctg aaaaaagaag aaaaagctca gataaaggaa cccaaaagct agcagataga

8701 cctcatcaat ttcgagactt aaataatcca agtaaatata tacttatgcc tagcgtttct
8761 tctgcccgac gaagatatgt gcctgttgga gtgtttgatg aaagtgtcat ttcgactaac
8821 ctgaattaca taattcctaa tggaacaatg tatgagtttg cgattctgtc ttcgctgatc
8881 cataacgact ggatgagact ggtcgccgga agaatgaaga gtgactatag gtattccgca
8941 tcagttgtct acaacacttt cccttggatt acacctagtg atgcacaaag aaaagagctt
9001 gaagttttag ctgaagatat tctatttgcg agagaagatt accctggaaa gactcttgct
9061 gagttatata acccagattc tatgccatta ggcctattaa gtgcgcatca gaagttagat
9121 acggctgttg atagactgta tcgtaaaaaa ccttttaacg atacttctga gcgcttagcg
9181 tgccttctat ctcgttatga agatatggta acaaaataaa aactaaagtt ggcaaaggtt
9241 tagataatga aaaacaatat cttacaggtt agttataacc aaacaggtca aagtgcttca
9301 cacaacgaca tgggaatgcg agaaatgcag gctcgtgcat tcgctgaacg tgctagccaa
9361 tatttactca tcaaagctcc tccagcatct ggtaaatcac gcgcactgat gtttttggga
9421 ttggataagc ttattcatca gtcggttcgt aaactgatcg tcgctgtacc tgaagtgtcc
9481 ataggtggct cttttaaaga tactgactta atgaagttcg gtttcttcgc taattggaca
9541 gtaaaaccag aaaacaatct gtgtatcagt ggaagtgaga gtggtaagac taatgctttt
9601 aagcgcttta tggagagtga tgatcaaatt ttagtttgca ctcactcaac acttcgaagc
9661 gtctttgata agttgttacc tactgacttc gataattgcc ttgttgctat cgatgagttt
9721 caccatgttt ctgctgatga aaatagccga ctgggtagtc ttattgatgt tttaatgaag
9781 cagtcatcag ctcatattat cgcgatgact ggctcttact tccgaggtga tacggttccc
9841 atattgctcc ctgaggatga agcgaagttt actaaggtga catataccta ctacgagcag
9901 ttaaatggct ataagcacct aaaaactttg ggtattggtt accatttttta tcaaggccgc
9961 tatgtcaatg cgttaccatc tgtacttgat ccgacaagga aaaccattat ccatatccca
10021 aacgtaaatt ctgttgagtc taccaaggat aaatacaccg aagtcgactc tattcttgag
10081 gtgctcgggg atgtaatgat gcaagaccca gagactggta tttatcaggt gaaatgtgca
10141 aaaacaggta agacgttgtt ggttgcaaac ttagttgatg acagtgatat gcggcctaaa
10201 gttcaggcat acctgcgtaa tatagagtca gcagaacaaa tggacatcat cattgcattg
10261 gggatggcaa aagaaggttt tgactggcct tactgcgagc atgttctgac tattggatat

10321 cgtagttcta tgacggagat cgttcagatt attgggcgtg caactcgtga tagtgagggt
10381 aaaagccacg ctcagtttac gaaccttatc gctcaacctg atgcacagga tgatgacgta
10441 aaggtttcgg ttaacaacat gctgaaagct attacaacct cgctcttaat ggagcaaata
10501 ctagcgccga gcattcaatt taaacctcgc tctcagtgga atggtgagga tttaccagcg
10561 aataccctaa tcgttgatga caccaccaca tctgtttcgc aaaaagttct ggatatcctt
10621 aatcggggca aggacgagat tctttcagca ttattatcta aagagtcggt tgttaaaggg
10681 gcgattgccg atacgatgcc agcagaggtc attaatgaga ttgaactacc gtctgtgatt
10741 caaacgttat atccagatct agaagaacac gaaattgaac agataagaac gggtgtgttg
10801 cacagtcttt atattggtca gaagggggc ttaattgatg gtaaagacct tcctgaagat
10861 gcggtgattg aagatggtgg tgcctcctat ggtaatggtc ataagtctaa agataatggg
10921 gaatcaggga cgagtaatca gtttataaaa atgggtgata agttcattaa tattgagagt
10981 ttagatatag acttgatcga ctcagttaac ccttttcatg gtgcttacga aatcctatct
11041 aagtccgtcg atgctgccgt gttgaaaact attcagcaaa cagttcgagc tagtcaggca
11101 aatgtttctg aagaggaggc tgttatgctt tggcctagaa tcaaagcctt caaagtcgaa
11161 caccaacgtg agccttcact aaactcctca gatcctatag agcagcgtta tgctgacgtt
11221 cttgcttata ttcgtaggat gaaacagcaa cgcatggcgc agcaataaga gggttatcac
11281 caaatgatcc gattacgctc ttctaaggct acagagaagt tcgacttaga tgatattttt
11341 tcagaacagg atgatttggg gctactcgat gtagctccct tgagggcaaa gattcccact
11401 gggaatatat tagccaatca atttgaagaa atttctaatt tctacgaaca aaatggtcga
11461 gtcccacgta gtgatgctca atcattttat gaaaagcgcc tagcgcgccg tctcaatgcg
11521 tttaagtcta atcctgaaca atgtgacgta ttatctgaat acgaccgtta tagcttgcta
11581 gatagcgatt tagccgcgca agagaatgta caaagagata aggtcgaaga taagtcttta
11641 cctaccgagc ttgataagtc tgaactcgta acttctctag atgatatctt tgacgatgac
11701 gacgatggcc ttttagagtt tgatgctcct cacctgttca ccaagaccca tgtgcctgct
11761 gagaaaaaat ctcagccaaa tgagattgcc aggaggcaac cttgtgctga gttccaccgt
11821 ttttccccta tattcgaaac gatccaacag gaaattagat caggctctgc atcactagag
11881 agatttcgtc atgaacttca gatgcgtgtt ggggatgtct ttatccttaa cggcctgatt

11941 ggatatgtcc attcagcagg cgaacggtta gaggggtata gtagctataa tgccagattg
12001 cacctgatct ttgagaatgg tacagaaatg catatgttgt tccagtcgtt gacgcatggt
12061 ttagttcgcg atgaacgcgg gtgtaagatt attcgtgaag gtgagtctct tgagcctgat
12121 gatacccctg ttccggcggg actcgtttat gttctagcta ctaagagtac agatagtgca
12181 ttagcacctt ataaggcaaa cctttataag gtcggcttta cagatggtaa ggttgaagaa
12241 cgtattaaat atgccgagaa ggataaaacg ttcttagagg ctcctgttcg tgttgttatg
12301 accaccgagt gttacaacat cgatgctcat aaacttgaaa cattgataca tggattctta
12361 gggcatcgta ggttaaacgt cactctcaaa gggcatgcag gacaatatag tccaagagag
12421 tggttttatg tgccgttgaa tactgtttta gaggttattg gctacatctt agatggaacg
12481 atatcccaat acaggatgga taacacgacg ggtaaagttg taaaaaaata agctgcttta
12541 agcctttgtg tagttgaaag cattatcgtg gttatgttct tagcataacc cgttttgatg
12601 acgtatgtcg gttaggaggg gcttttagaa agagcagaaa gtccccaagg ccgctaggag
12661 gcgttattgt ctgtagtaac aactcatact ctcacatccg ctgttttgga cacgattaga
12721 tactgtcagc ctgagggtat atcaactcgc aattggatat ttcctagttg aaccattgct
12781 aatgtagtaa agcctaaatt aagtaaggta tggcaaatta tccgttagtc tagtgtcaag
12841 agtcacaaga gaacatcaac aaatgaatgc cttaagtgta ttagttccga ctagatctga
12901 cacttcaatt tgaaaagaag agagttaccc actttgatta aaggtgctta atcactaatc
12961 aaagacaaaa agaggtaact ctcatgcttc atactagcaa tccaattatc aaacacaaag
13021 cgggccttct caatcttgca gaagaactcg gtaatgtatc tagagcctgt aaggttatgg
13081 gggtatcaag agatactttc taccgttatn caagagttgg ttgagacggg gggtattgat
13141 gctctgatta accgtagccg aagagcaccg aatttgaaga accgtgttga tagtgaaact
13201 gagcaagccg ttatcaaata cgccatcgac ttccctgctc atggacaagt tagaacgagt
13261 aatgaattac gtaaattggg agtgtttatc tctccaagtg gcgtacgctc aatctggctt
13321 cgcaatgacc tagagaactt caagaaacgt cttattgcac tggagaaaca ggtcgcagag
13381 aacggtatta tcctaacgga cgagcaagtt gcggctcttg agcgtaagaa gcacgatgat
13441 gaggcttgtg gtgagataga aacagcgcat ccaggttatc tcggctctca agacacattc
13501 tatgttggca acttgaaagg tgttgggcgc atctatcaac aaaccttcgt tgatacctac

13561 agtaaagtcg cctttgccaa gctctacaca acgaaaacac caatcaccgc agcggatata
13621 ttgaatgata aggttctacc gttctttgag gcgcatgaac tgccaatgct gcgaatcttg
13681 actgaccgag gcactgaata ctgtggtcgt gttgagcagc acgattacca gctctaccta
13741 gccattaatg atatcgacca cacgaaaact aaagcgatgt caccacaaac aaatggtatc
13801 tgcgagcgct tccacaagac catattgaat gagttctacc aagtgacatt cagaaagaaa
13861 ctgtatggtt ctatcgaaga gttgcagaaa gatctggacg aatggatgga ctactacaac
13921 aatcaccgta ctcatcaagg aaaaatgtgc tgtggcagaa cgccgataga aacattagag
13981 gatgggaaat caatctgggc tgaaaagaat ctagcccaga tataatctga caggcaccaa
14041 gtcgaaaagt gggtaactgt cagatcaggt ctgaattagt acaccttaag tagtcaattt
14101 ttcagatttc attctagtta aaggggttat tatcgttttt gtgcccctca ctgtgaacgg
14161 ttgaaccaat ggcagataaa gatgcttctt ctgctgccag ttctggaaag tccgcttctc
14221 tgggatcgtg gtgaatatga aggttatcgc cctgaggtat gggctgattt gtttttttcac
14281 acacgttacc taaaccctgt ataactcttt gattcttata atcttgcttt gccttggtag
14341 actttgtttt caccgtctct gatgcaatgg cccgctcctc attaagctta ggcatcatat
14401 tgattttatc aataacttgc tcaaattgct gagcttgaga acgcacgttg gggtcaacat
14461 ctctcaactt agggtcattc agtttcaaag cactagaaac acgaattaag gtttgctctt
14521 gatcaatgat cacttgctct tcagatgcat ttgcgacatg ctcgttcact tcaccacgag
14581 tcaatggaga gttttcaacg agatcgttaa cattgataaa caattgacta tttgtaacct
14641 tagtatctgt atacacttgt aatacactaa atccttgtcc attcacggaa agttttttttg
14701 tggtatctaa gtgatattta aacatgggca tttttattca tcaccttgaa ctttgttgtt
14761 taacagcgca attacctgtt cataatctaa cttacctacc tcattcacta tacttcgtgt
14821 tgcctcaatc cgcgctcgct ctccgcaaga acgtggttgc tccgagcgct cttgcaaaaa
14881 ttgtttggta agatcgctgc gaataaggta tttattattt atttttatta cctcaccatc
14941 tgccgcatct gccattaact ctctgaatgg ctttctatca gttccaatgc atgacactaa
15001 tgaatcaaca ctaaaaaatg gagtaggtgt tttggtctga tgatcgatct tcataataga
15061 cttacttact aatacttgat tgttttcggt aagtttttca tccaaatcgg gggagatttc
15121 gatgggtttc acgtcaatca attccgatag tggcttaagc tctatgggta aatttgtgtc

15181 actcatgaac attacccact aatatgtaac tttgttatag agttgcttta
atttatcgaa
15241 agcctccgcg gcccttggat catcatttt gtctggatga actttatttg
acagtgactt
15301 ataccatttt ttagctgcat cctcgtcctc atacatctct gatgtgatac
caagcaattg
15361 gtttcgacgc ttaccatcaa cctctaacaa caaaaacaaa atgcgatcgt
tttccgactt
15421 gaagtacgcc atatcatcag ttgatgtcct gacaagatta acctgtagat
cagcctcttc
15481 agcggtcgta ctagattcaa cataggtgga ttgtaattct tggatttttt
tcttgtattt
15541 tctcaacaag aaaagaagtt gcacaactgt taataggagt gcaaaaacaa
caacatacag
15601 tgatgtcata tatatttcct tggtagtctg aaaagtatag gaatgtttta
taccacatca
15661 cctcaggctt gcataagtga gacagttagg aaaggcatat cacattgatg
aattcgtgtg
15721 gcggatgaga aaatgtggcc acttagctag agcatttgtc ataagaaaaa
gtaagtcaaa
15781 gaccaaactg aatgctatgc acgaaagcaa cctaacaaac aatggggaat
gaacgtgcca
15841 gaacggatcg gcctgacacg agtagggaca aatccttgtc agtgactttc
ctaaattgta
15901 ttagttccga ctagatctga cacttcaatt tgaaaagaag agagttaccc
actttgatta
15961 aaggtgctta atcactaatc aaagacaaaa agaggtaact ctcatgcttc
atactagcaa
16021 tccaattatc aaacacaaag cgggccttct caatcttgca gaagaactcg
gtaatgtatc
16081 tagagcctgt aaggttatgg gggtatcaag agatactttc taccgttatc
caagagttgg
16141 ttgagacggg gggtattgat gctctgatta accgtagccg aagagcaccg
aatttgaaga
16201 accgtgttga tagtgaaact gagcaagccg ttatcaaata cgccatcgac
ttccctgctc
16261 atggacaagt tagaacgagt aatgaattac gtaaattggg agtgtttatc
tctccaagtg
16321 gcgtacgctc aatctggctt cgcaatgacc tagagaactt caagaaacgt
cttattgcac
16381 tggagaaaca ggtcgcagag aacggtatta tcctaacgga cgagcaagtt
gcggctcttg
16441 agcgtaagaa gcacgatgat gaggcttgtg gtgagataga aacagcgcat
ccaggttatc
16501 tcggctctca agacacattc tatgttggca acttgaaagg tgttgggcgc
atctatcaac
16561 aaaccttcgt tgatacctac agtaaagtcg cctttgccaa gctctacaca
acgaaaacac
16621 caatcaccgc agcggatata ttgaatgata aggttctacc gttctttgag
gcgcatgaac
16681 tgccaatgct gcgaatcttg actgaccgag gcactgaata ctgtggtcgt
gttgagcagc
16741 acgattacca gctctaccta gccattaatg atatcgacca cacgaaaact
aaagcgatgt

16801 caccacaaac aaatggtatc tgcgagcgct tccacaagac catattgaat gagttctacc
16861 aagtgacatt cagaaagaaa ctgtatggtt ctatcgaaga gttgcagaaa gatctggacg
16921 aatggatgga ctactacaac aatcaccgta ctcatcaagg aaaaatgtgc tgtggcagaa
16981 cgccgataga aacattagag gatgggaaat caatctgggc tgaaaagaat ctagcccaga
17041 tataatctga caggcaccaa gtcgaaaagt gggtaactgt cagatcaggt ctgaattagt
17101 acacctaaat tagcatttaa ctgattacct cagacattgt aaactctaac tgcctcgccg
17161 aattcactta tccactacag actgtgttga atatagaatt gccgaaaaca tcatctaata
17221 gttcgttgcg ctccttgctc atataaccca tcctctgggt taaaaagatg acacttcttc
17281 atactcaaac aaccacagcc gatacaccca gagaggttat cttttagtga ctggatcttt
17341 accatttttt tgtcaagatc ttgctgccac ttcttcgcaa ctctatccca atcttgttta
17401 gtagctgttt tgttcatagg taaggtactt aactcatacc ttatttcttc tagggtaaac
17461 cctactgact gtgccacttg gataagtgcg accctacgta acatagaagg caagtaacga
17521 cgttgattac tattggttcg aaaagatgtg atcaactgtt tggtctcata aaaacgtaaa
17581 gccgatgtcg cgacaccact gcgttctgat aactgcccaa cggtaaggta atcatttcta
17641 atcataatta ttctaacttt tgtcaattta tcgctttact taaagttaac tttaactttt
17701 atactccttt caaatataag actcaacgca taaaaggata tgatcatgcc gaaatccccc
17761 cccataagtg aagagatgcc aaattgcatg tcggcagata cgcagtcaat aagcaactta
17821 attcctgtac ctaaactagc gctggatagc ataacaattg atgttgatgc cgttactttt
17881 aaatcggctt agccagtacg ccataatctg tttcaacttt tcatacagct caacctgttg
17941 agctgcgttt ggattcttta ctatgcttaa aaactatttc actttgatcg caattgggct
18001 gatttgggga tcacaattta tttttcaaga aacagcacta gaagcattca ctccagtatg
18061 gataggcaca tttcgtgcgg tactcggtgc cacaaccctg tttattattt gtcgaataat
18121 gcgaatttgc agtagtagta aacagtggaa actctacact gtaatcggtt ttcttgaagc
18181 aactgttcct ttcgttctcg tgccttttgc tcaaaaagag ttgagtagtt cagtgactgc
18241 aattttaatg ggaacattac ccttttacgc acttcttctc gcgccattat ttattaggaa
18301 tacaaagatc accaaaggaa acctgttcag tataaccatc ggtttttttg gtttagttgt
18361 actttctac cctgacctaa tttcatcatc tcaaaggatt gagcttgtca gcgtgctagc

18421 cgtcttactt gccgcagtat gctttgctat tgctttgcta ttgctaaacc gtgttcaaag
18481 agagcatcct ctgatagtcg caagaaatgt tctgaccatg gcgagcattc aactgatcat
18541 tgtcgctctc attacaacga cgagaatgac atataaagca ccatcgccga gcatttttat
18601 tgctgtaatc tatcttggtg tcatgtgtgc gggagttgtc tattacttat atatgactag
18661 cgtcaagaat gcaggagctg tttttacgtc aatgactaac tacctcgtac cagctgtagg
18721 tgtattaatc gctgcgctag tcacgaatga aagcatacag actacaactt ggctggcact
18781 tggtattatt ttatcagctc ttttattgaa tcaaatggtc gaaaagtatg aacaaaaaac
18841 ttagcgggaa tgttcaggct atagttgtaa ctttttagta aaacaaaacg ctcggtgtac
18901 ttcatcgggc gtgtgtactt taaagcctta tctaatctta cttgattata ggttttatta
18961 cccatcagtc taacttgctt caaatgtctt aggttcaaat agtaaaactg aaagcgttac
19021 acttcaaaag agatacctat tagttttgat catgaattat aacgcatccc gaaacaaact
19081 ttggggcaaa aactagttag ggtgtttaag taaaatagcg ctgtaaggat taggagggat
19141 aatgaaaaca taccgttgca gggcttaaaa acaacaaaga gacaatttaa aaattatctc
19201 tttgtaagtt gggctacaag agagtcatac ttttaaagtc ttattatgca tattgtagaa
19261 atcacctaca caatattcac tgtgattaag gtcgctatca atcatagatg tagtgaaaat
19321 gatttggtgc tctacatcta gagagttgga tagtttcacg atctcacgct ggaaaagttg
19381 gcttcgttct tcttccatcc ccttatcttc aatattgtcc ataagtaaga agcgagggta
19441 caaataagaa gagtcttcac atgagcactt aagcatagcc aatctaaatg cgttctttag
19501 ataaaccatc gaactagcag aaaacaaggc tctatcatca atggtcactc gatcttctgc
19561 aaagtcgaac tcgactttgc taccatcaac gaatatttct tcatgtccag cgtcagcttt
19621 aagaatactt aacgttgctt ctgatagctt tctcttcttc gctttaagct cattttccat
19681 tcgctgcgtc aatctagtga tttcatcatt cagcgtattg aagtctgtct gcaacgtatc
19741 tcgttcatca tataatccat agagtttagc gaactctttc ttactattgt tcagtgttct
19801 gatttcattg cgtaaaccac ctatttcact gagcttttgc cttagctgta tattcaccgc
19861 tctttgaggt ttgcggatga tctcaaggtc tcgttccaac tcgcctaact tagtttcaag
19921 ctggttcgat tcaaccttct gctcatcaag ccttatttgt tttttctcta tcagaagaat
19981 tgactcggca atctggaact ctatttcttt tctcacttta aatgtagggt cgatattatt

20041 ctcagattca ggttttgttg caccacacaa agaacaacct gtaggtcttg cttggacctc
20101 tgtttggcaa gctgggcagt gattaaatgc aacgtcagat aaagcgttga tagtatcttg
20161 ggtttcttta agagccttaa ctctacggct taaagactca ataaacatct gagagtcatt
20221 gatttcaaaa gacgttttag ctatttcttg ttggcttgct gtgtactttc ctcttgcctc
20281 ctgaacctct acaatcaaat ttttaagtac ttcatcttct gtacaaccat cttccgatgg
20341 gctagaaaac gattcaacct ctagctccag cgcatcaatc tctttacgct tttcttcaat
20401 ctcaccattt atttcactaa cagattttat cgtaccaccc aaaaatgagt gtatggtttt
20461 aatctcttta attcgatttt ccaacgctag tttcaagctc tgtaatctga cccgtttttc
20521 atagagttca aaatctgaca agccgatcaa tagctccccg atagcttgac gcttgtttgg
20581 tgaatcgaac ttatcaaact tgaatagtcg atctggtgac gtcatctgat caacatagag
20641 cattctaagt aactgatgca ttgtaatact gttcttgtca tccgactttg aatacggcat
20701 accaagctct ttgaacaagg cttgataaaa actttctttt gtattggcgt tataagggta
20761 acgaatccaa tcagtaacag atgcaacgta tgctgattca tagtctccgc taaatatatc
20821 catccctttc cttccaccct ccgcgatttc ccgctttagg gtgaatacct tgccgcttag
20881 agaagcttcg acaaaaacga agctacactg cttcgcttca ttcttaagct tagttaaatc
20941 accgccaagg ccgtagaata taaagtctga gatcgtagac ttaccagaac tgtttacgcc
21001 tctgataata ttcacaccag cagtgaactt ttcatcatag gcaatacttt tgcctttggt
21061 aatgactagt cttttgacta ataagaaagg tttatactgc gtcatatctg tattccatta
21121 agcctgttct tttcttaagt ccactttcac caacaatctc aatcgcattt agggtttgga
21181 atagcttgca aaaaagctcc gtagactcat tagagttatc gttaatcagc ttttgaatag
21241 acgggctata gaacccctct gagatccgca agttaccgtt ttcttcctca aaaataccct
21301 ttgcttttag tatttgaagt gcctgtatat ggtagtcgcc catctctgaa aatagtcgtt
21361 tcttatctgg tagaacttcg tatggttgat ttattgagcc aaagctacgc ttcaactcag
21421 cgattccttt agttcttgga aactcaatat cttttacaag gtgtggaaat aggtaaaaca
21481 aatcgtaaat acgcagtctt accgagtcaa aatcctctaa atcttgcata cttgctatgc
21541 tgataaacct aaacatacag tggtaaagat cgtttcttgg atgataaata atcactttta
21601 atcccactct acatagcagt tacctgtaag tatatacagt agtccgtaca gctcatcttt

21661 gtcaatattg aggctgcacc catgaacact ttccaataca ggttggatga tttttcttc
21721 gactaaatca ttgatttggt agactttaaa gtcgccagat ttaatgcgtg gtgaaatctc
21781 gtggagaaac gtggttctga tattagtgag gatatatgtg tagagttcct gagccgattt
21841 gtaatgcgag aactgcatta tcttcttcga tacacgctct ttgacatcca tagcgtaatc
21901 caagagaaaa tctctgttac catcggtgag cttttgccct agatctcgaa gcttcccttc
21961 gacttttcgt tccagaaagt tattgagctg actagaaaac tctttgtact gttcatcttt
22021 ctgaatttct ttttcatgct cttcaacaag ctctcgcagc ctatgatctt gacgatacat
22081 aactggagct gcgtgattat atgttatgtg ggttgagtga tcatgaacat ctcgtccagc
22141 gacactacgt ccagcattaa cgttgtcgaa agaggcttta tttgaatcca gtaccgacac
22201 ttatgaccct tttttgtccc taccaacaac atcgcctcca gcgcgaatat tgctcattgt
22261 tacattgttg gaagatgctt ttgacctaaa gaagaaataa cctataactg cgacaatagc
22321 cgctacgatt aggctaacaa cgtaaacacc taagccagag aacaaaacat cgaagttttc
22381 gcagacccag tttatagctt catccaccct aacatcctcg tggtttttcc ctaaacttta
22441 caagtattta attttattca caaatcgaca agccaaaaat atctacttac ttctcaatat
22501 atgcaatcgg ttaggtcact atgtagatgt atggctgaat cgaccaagct tctttgaaca
22561 ttgcactgat tagaaaaatt acttcaaatt gtacacacta gatatgatgg attcgcagtc
22621 taaatcgaaa tggggcaaaa ccaagctagt tctgtattag atagagcata attcacacta
22681 tctcagcatt tgatttttgg taaatcttgc caacgggtgg tgtattgagg cgacaacatt
22741 tcacgccgca ttgcccattt ttgagttatg ccttgagcag caagaaagat agcatcattc
22801 ccaaatcgat tgtttaacgt gtcatacaca ttcattagct taggattatt cggggtcagg
22861 ttgaacagat cgggctgctc atgctgaccg tcaactaagt caatcagccc aacaccaatt
22921 ttataaaatc tcacatcctg ttggaataac tgctctgcca gtcgagaggc ggcttgagtt
22981 atcagagtca catcagcagt aggatacgca aaacgatgaa ccgctcggcg tgccacagga
23041 ggcgagtcaa atggtgaact gttagcgaaa caaagcatca ctcgacacag tgatttttgt
23101 tttcgagcct taaaggatgc aatatttgca tgcttgcaca acgcttgctg caacgactct
23161 aggtcagtaa ttctctgccc tgcactgcgg gtagaaaata tttgcttttt atccgctcta
23221 gccgcatccc agcccttaca tttttgtcca ttgagttcac gtactgtacg ctccacttct

23281 acgttaaact cttttctaat cagtgcagga gggtaattgg caagcttgag
cgctgtatta
23341 atacccatca tctctaatcg atgggttagt ttcctgccta tgccccacac
ctctgagaca
23401 tgcagttgcc ctaaaaccgc cttacgctct cgttcgctat ctaagacaca
tacgccattg
23461 tatccttcga tttttttcgc agcatgattg gccacttttg ctagcgtcaa
tgtttgccca
23521 aatccgacac atacgggtaa tcgacattca cgccaaacag cgcgtcgtaa
tttcattcca
23581 tgctctttga gagaacgaat ggctggaaag ctatgctcaa atgaaaggaa
agactcatcg
23641 atactgtata tgtgttgttc aggtgcgaag cgaccaataa cctgcatcat
tttggcagat
23701 aaatccgcat acaactcata gttcgatgac agagcaatca cacccttctg
ttcacaaagt
23761 gctttaactt gaaagtatgg tttgaacttt tctatgccaa cttctttagc
ttgtcgattt
23821 gctgcgacaa tgcaaccgtc gttgttggac aaaacgatca tcggctttcc
acgccaatcg
23881 gggcgaaaaa cttgctcagc gctacagtag aaagaattag catcaaccaa
agcaaacatt
23941 atctcgttct caacaatgga ctcactcggt ggcaacgaat tgagcgagtt
acaatccctt
24001 cgagggaaaa ttgatcaaat tcgtggatag gaacgggctg gtatcgctca
ttagcagaga
24061 ggagtaatcg acgattgagg tcgatttgtt tacaaataaa ttccccattg
aggtttgcca
24121 ctatgatgtc gaaatcttgc acatctaatg agcgatctac aataagtaaa
tctttgtcga
24181 agatacccac atcttgcata gaatctccac atgcgtatcc cagaaaggtg
gcacaaggat
24241 gctcaataag aagctggtct agatctaaag cgagttgagt atactcttca
gcaggactct
24301 cgaagcctgt gataccagca tgagcaaaga tagggatgac tttcatacaa
aacatatgct
24361 gtatattgat acagtatatt gtatttcaga ttaggcattt tgcaacagca
ctgttgagat
24421 aggatttctg agcttaatat catcaagaga actattgatt gacaactgga
ctcaatatga
24481 gttgtctcaa tgggatgatg ctcgtacatt cttggataat tccgagttac
agtatttgct
24541 aaattagtag ttagcggacg aattgaggta tggcaatacc ttctctacgc
agaatggtct
24601 accttgaggc gcgtgtctac agatatggtg gtgttatact tctccatgaa
ctaatagggt
24661 attgccatgt actaatggga gggtttttat ttttaaatac ctgtttttta
tcagttgttt
24721 aaggtttatt tctcttttct attttctaga aagattatgc gaagacatat
ttaaagacag
24781 caatgcttca tgtaaaaagt actcatgttc tacgtatttg aaggttttca
ggaggtagtg
24841 atgttagagc tgtttttaag tagtttggag agggtcgcta aaatacaacc
tcagataact

24901 atcctcattc tagtgttagc cttattaggt agtgacttga tctcgttcat caatgacttt
24961 atggcggtaa tgaaatgact ttaatgctct ggtctaaaat gccctcaaaa tggattgcag
25021 agggaatact aagagagtcc tttagtggtg gtaggagtgt tagtgatgat atcgctgctt
25081 tgaagttgtt cattgtactg tgttttcttg ccaagcaagt taaccgttcg agtggtagct
25141 cggtggatga tgttttggaa atcagggcaa catatgatca gcttacagat atgtgttctc
25201 tgagccgttc tttggtgtct agaggcttaa agaagttaca tataacaggg ttgttatcat
25261 ccgctggtgt tcgaacaaaa acatatactt ttattcaagg agtctaccga ggatggtgca
25321 agcttccaaa aagagcgctt gttaaaaatg aggaagaaat cccagcgatg aaagcttttc
25381 tcaataggta ttcccacgaa cgtgatgctc taaaatgctt tttgtacata cttgctagtc
25441 gaagcaatag tagaacttac gtcgatctta gtcggggaac tatagccaaa aagactggcg
25501 taagtctgga tgctattgat ggcgctatcg gatttctgca aagcactagt ttgatttcta
25561 aagtagagga caaaggcttc ttggctaact ctattcgtcg tgatttgtca gaacgactgc
25621 ataggtactg ggtgattggt agcagcagtt tgaactacaa gacatattcg gttgaaagtg
25681 aagactctat ttttgttagg gaaccatcgt tgtactttat ggagaacaac aggagataat
25741 gcatatttca ttctcacgta attcaataat ttatctatta attcaatatg ttaattgacg
25801 tattgagagg ttggactgtt cagtgataca taatttctag atgtgctgaa ttcagataac
25861 ttttggtaag gtaaaatcgt attactagaa tgtgcatgag tatttgctct aagtgaaaat
25921 ggggcagaac cgatttaggg ggacttgtca gatctatcca tatcggggaa acgaaattga
25981 cagattgtat tcagaccaat tcaaataaac gcctgtttag ctttttttcag cctcaatcca
26041 gcttcaatct ctgcaacatg ttcagctcct aattcggcaa ctaaatggtc acgagttact
26101 ctatcaaaaa cctcactttc atcctcatct tcataaagtt cagcttccgc gagttcgaca
26161 tagtgaagca ttgctttgat actcgcatgc ccagttaagc ctttaattct gtttaggatt
26221 aggattgcga gttgagtccc gccgatgttg gtttttagag ccttaagttg ttgtaataca
26281 agtagcgtaa taaaccgatg cctaaacagg tgggggctgc gtttatgttt gctcttaaac
26341 ttccgactga atacatcata gaataagcgc gaaatcatta ttccactcat cggcaagtga
26401 gagcttgggt gcacgaataa cttcccgaat tttgcacggt taactttagc aagccctttg
26461 cttatgcgat caagttcatt aactatatag tcatcataaa accgaatgag gtattgtgct

26521 gttgtaaatg aaatcggaat aatcctaaat ttatctttgt tcttgccttt ggtggtttta
26581 atttttatcc aaataagctg gtcgttagcg ctgcctaaag ctgatttacg gtaaatagct
26641 tgagctgcct gtaagctctg tgtattgatg gtaagcttat ttagactaat aatttcatcc
26701 agtcctatag ccttaccact caaagatgca tttacctgcg cacgtaagac ttcaattgta
26761 tgagtgtcaa tgtttgcggc ttcacttact cgaatgcctg ttgcctcaag caaactaatt
26821 agagtagaaa agaactcaaa acgatatagg tcaccttccg cttccaagat atctaggtca
26881 tcatatagac tttcgattgc agtatctgtt attggacttc tactaccata gctctcacag
26941 tggggtatgc agtcgtgatg caagtaacgt ttcccataag gactgatttt tctttctgca
27001 ttaaccaacc cctttgtaaa gtattcacca tcagtattag ctatagctat taacgtatag
27061 ctcaaatggt agtatttttg tacatattca agaaaccgta gtgccatcct aactctgtta
27121 gctatagcat ttcgatttag gccaatatcg tcttcgatat attggacgta gtcatataaa
27181 tgagagtcgg taataaggcc aatagggaaa gggcgtcctt gaatatcgtt atatttccaa
27241 ttttcataac attctaggta ttcaagaaaa taccgaatca tacctttggc agttttcttt
27301 gaacggttcg gtattgcatc tgacattaaa aagtctgtcg caaaccaatt aacctcacca
27361 ttaccatgca aaaacaaact tgattcccaa ctgatgttag gatcactttc atctgagggc
27421 agacagtcct taacatctat ttttaggcta tctaatttca tatgttattt gccaaatatt
27481 gacacaatat tttcttcact ttcagcgaaa atatcatcaa attcatgaga gagtttgttt
27541 aagtttgaaa ttgtctccct tagagcgggt ttaataaccg ttctaacttt acatagttcg
27601 gtctggtgat cctcgtagta tgatttcaac gtccgtgaag cgtatttgtc acgttctttt
27661 attaaagata ttaaagtgtt gttagcactt ctttgccgca atatctccct agctaacgat
27721 tctataagtt ctgcttgctc tttaagggca ctccgtagtt cacctttagt tttaactttg
27781 ggatcgtcgt cattattgca ttcattctct gcttcagtaa cttgctcgga tggagtgctt
27841 gtagtttcaa tcttgcttct taattcttct atttttagga gtaggtagtc cttacagtca
27901 accactgatg aagcagagcc agtgaaggat ggatctattt cttttactag gatttttttc
27961 ttattgtaaa tggcaggata gctacgtgat tgtattttgc cactatcaga gtatgctaaa
28021 agtgctttta aactgatatg ggcggtaaat ccatctggca ctccattctc aataaaaatca
28081 ttaagaagga ttacacgaag tttaacctgt tcaagcgtat ccctccgccc tttagggccg

28141 ttgtggtttg gcgataattc ccttttaaga ttcatcttca ttacccacct ccatccaaac
28201 aattaccttt cctgattcat ctgtgtagct atatatcttc gagccagata gaagttgctc
28261 ttttataaat tcttttgcca actcatctga ctgattattg aatttaatgt ttagctcagt
28321 tttgattaca ttttccactt gtgatgtatc aacacctttt ttgtaaattg catctaatac
28381 accctgtgct tcttcttctg atagctcttc gttacttacg tcctctagca agtaacgcca
28441 agtttcctga gtgctgcggt gccccatgag ccaagatatg gcaggaaaat cacttatatc
28501 agtaagcgca tagaaggcac gagcagcgaa tcgtcttaaa acatgggtac gagataaata
28561 aaagcgtgat tcaataccat tcaccatttc tgttggaact tggataaagt cgccaaaatc
28621 tttcagtatg ttactcattg caaagcgact cattttactg tttttttgggg gcagaccttc
28681 cgctgctgag gtgtattttt caggaaacaa aaaggggtct tcgttttcac aatagtaatg
28741 ctcattcgct tcagccagat atgagatccc ttcaaataaa acatgaggta taggtcgtcc
28801 agtaacaaga atggaattct ctgtaggcgc agcctttcgg ataccaaaaa acaactcata
28861 gccaccccaa aatccgagtt ccaaattaga ttctttgagt tcaagtatct ctgttgttcg
28921 cttaatgcaa aaggtatgtg ttagaatata tgttgctgca agcaatatcc tgtatccgaa
28981 taaaacagtg acattttcac gtagtttctt tggtgtagat cctttctgta actcattata
29041 tctagttacc ttgaaatctt gagtaaactt gtttttaggg atggctacat tcactgggta
29101 gcgtccttca aaaatatgag aacggagata acttggattg ttcttgtgga ttttatttag
29161 ctgtaattca cagtttatct ttgttgcaac aatactttct ccgtaaacaa ccacaatccg
29221 aatagcttca ttcaaataat gtagagctgt tttggtagga atattaggag tgcgacccctt
29281 tgatctgatg tgattttttt taaatgattt actaaccttt gcaataggat tgcaataatt
29341 ctccagttca ggtaaatagt ttgaatatct ctttaaaagc aatggtcctt tagtcagagc
29401 atcaaaagta ctcgatgaaa tattattttc ttttcctgta aataactttg ggcatgaatt
29461 aggaaactca gatttttac cattattatt gccagtgatt gtcagtgata aatcaggaaa
29521 tcgttccact aatctgtctg aaattaaatt cattgtaaag tctgatatac ttgtgttctg
29581 cataccaatc ctttgtaaaa attcacctac tggtaattcg ttgtttttgg attctacatt
29641 tatgtcaaat agttggattg ctgattctct atccatagtc gatatttttt ctaaaataat
29701 atcagtaagc cctgtagctt tgtctgccct ccctgtggca atttccaagt gaaattttct

29761 tatatcggtg gtagtcagaa ggttgaatcc ttcttcggat acagcacttt caccgtactc
29821 tttaattaag aatagagcta atattttgag cgcgatggca ttgctattca tacttgaaaa
29881 gcgtgtcagc aatgacgaat ttttggtctc tgttgataaa tgtcccgttc tatttagagc
29941 aataactttc cttatagtgt tagttactat caggtattta tcctcatcaa gagtatcacc
30001 tgttgtcgga atgtaatcat taaacgaaat atatgggagg tcggaaccac tttttcctga
30061 accattatca atatgccata ctgagtctga aaaattgcca tgaagccaag caaagtgcca
30121 ttttgaggtg tcatctgatt tataagacca gtcagcccct tcacgaagct ttacgttatt
30181 gatattaatt agagtcatac tgaacctctt gctttattat ttttagatca agcaagcttc
30241 cagttttttt ctcatatagt aggttaccct tctgcaaagt tcgcattagc ctcctatctg
30301 gatgtgtgtt caagtatccc gtcaacccct gatacacgaa tgatagttct gaaatgctgt
30361 cttctgtatg attggcagaa tctatgcgct gcattgctat ctcatgacaa gccttcatta
30421 aggctatatt ttcaggacag atgtctagag taattttaat atcactggct gtactggaat
30481 tcctattgtt atcagatata gtctctatca cgcttctcca gtgaggaatg cgctcatcta
30541 gccgagataa atactccgtt agctcagttt cgtctttcag cttcagagcc ttaatttggt
30601 atttttgtcc atcagtagcg ctgctgatca atacatgctg gaaccgcctt actcgctggt
30661 tataaaccgc caactgtaac tcttggggta agtaatttct tatggacaca gcttcagagt
30721 tcccaagata tttagctgca acagcagggt ttccaccact ttcataccaa cgaatgacac
30781 cctcagtagc acggagcttc ttggctgttg gagctgccat tagcacttca tcaatacatt
30841 ctggcgttac tccttcacac caattagggg tgtttttaag gatctttttt gcttcgacaa
30901 gtaggcgaac gaaaccactc ttaaaagcca tatcactcat tggcattact ttatgagttg
30961 cagtagctgt ctgatacaaa aataatctat tggcatcatt gcccgatgcg agagagcggt
31021 gatatgaggt tgcctcaatg atgtagttaa aagccttttc aatgaacgga gtatccgttg
31081 catgcctcaa attgcgcttt aaatgctctt tagctcttgc tttatggtag ctgactcgga
31141 atccgtgttg agatggttcg aaaatcttgt taatcgagcc ttgagctgac aatgctaatt
31201 tacggagact atcaacgtta atacccgatt cggtgatgat tatgttgcag caagcgacgg
31261 ccgataacgt ggacaaacca aaatgctctc ttaactcata ttgattactt gtgaatctgt
31321 aaagatggtt gttaccgccc aaaaaatgac gatcaactag tccattttta caatgtccaa

31381 tgtatgctag taagttggtt ttcccacatt caccgttagg taaatctgga ttaaaaaggc
31441 tgagtcgaga gccttttccg tttaaaaggg agggatgaag tagagtgggg ttattttcaa
31501 acacactagc atctattttt tccattgcct cttgcccaaa ggtaaaccta tcgcaagcat
31561 caatcagata gcttttagat actttataga tcacgtcacg atgctttttc atcgtggata
31621 tcaaattacc tagaaaggca tctacatccg tttcaagtat taaggtgagg ctttcccccg
31681 tagtgttagt tgataactga aaacctccga ggactttggc ttgggagttt gtagtatcag
31741 agaaaactaa agtatttaaa cgtcttgggg gagtgatgtg gttggttttt ggtaggccat
31801 taactgaacg tttacaaagt ctcaacaaag cactccaaag gcttcgggaa tcactcagca
31861 aggtgtgtgg ttgtttcttg acagcgccgc taaaatagaa ctcttgccat gcagagtagc
31921 ataacgatac atcagctccc gtcttcatat gtaggttgtt cttataacag aatgtgaaga
31981 attcgtttaa catcctcgtc gtacgtcttt tgtaagagtc tgatttacca gagagaattt
32041 tagtgatgga tatgctcata tttacagcat actgatgtgc catgattttc tggcagtgca
32101 taccgtttat tgtatgctct tttatgttca taaacacttc caccgtataa tgtcattgct
32161 aatactttga aagatattag cactaaggag tgttgtgtaa agcattgttt tttatactta
32221 tttttatgtt agtgcatgag gttaaaagaa cgatagtgga agattttgtt caatatattg
32281 agatgctgaa gttagatgag gacaggctcg atttatttat tataaccaat gtcagtatgg
32341 atggggggaa gtgtgagttt atcctaacta tagattcacc agtgcctaag ctgtcgagta
32401 agtggttaat ttcatgtgag gattgtctga agctacatat agatagtacg acgagtccag
32461 ctcatgacat tgccataaaa tacggcattt tagttattgg aacttcgtat gtaacaggag
32521 cctatttcaa agccgtacga ctaaacgttt aagtttttat tcggaagtgt agcattttta
32581 tttggttctt catttgtggt gacgtgcgtc gatttctact tgcctacaat aaatcctacc
32641 ttataggtgt ttcatttctt accttataat tgtgttttgg tttcttacga gaacttattg
32701 caaactacgt agttaatgat atgatcttta aggataatct tttattttg ttttaagtta
32761 cagttttgtg gttccgataa aat
//
